# Critical domain interactions for type A RNase P RNA catalysis with and without the specificity domain

**DOI:** 10.1371/journal.pone.0192873

**Published:** 2018-03-06

**Authors:** Guanzhong Mao, Abhishek S. Srivastava, Shiying Wu, David Kosek, Magnus Lindell, Leif A. Kirsebom

**Affiliations:** 1 Department of Cell and Molecular Biology, Biomedical Centre, Uppsala, Sweden; 2 Discovery Sciences, AstraZeneca R&D, Cambridge Science Park, Cambridge, United Kingdom; Max-Planck-Institut fur terrestrische Mikrobiologie, GERMANY

## Abstract

The natural *trans*-acting ribozyme RNase P RNA (RPR) is composed of two domains in which the catalytic (C-) domain mediates cleavage of various substrates. The C-domain alone, after removal of the second specificity (S-) domain, catalyzes this reaction as well, albeit with reduced efficiency. Here we provide experimental evidence indicating that efficient cleavage mediated by the *Escherichia coli* C-domain (*Eco* CP RPR) with and without the C5 protein likely depends on an interaction referred to as the "P6-mimic". Moreover, the P18 helix connects the C- and S-domains between its loop and the P8 helix in the S-domain (the P8/ P18-interaction). In contrast to the "P6-mimic", the presence of P18 does not contribute to the catalytic performance by the C-domain lacking the S-domain in cleavage of an all ribo model hairpin loop substrate while deletion or disruption of the P8/ P18-interaction in full-size RPR lowers the catalytic efficiency in cleavage of the same model hairpin loop substrate in keeping with previously reported data using precursor tRNAs. Consistent with that P18 is not required for cleavage mediated by the C-domain we show that the archaeal *Pyrococcus furiosus* RPR C-domain, which lacks the P18 helix, is catalytically active *in trans* without the S-domain and any protein. Our data also suggest that the S-domain has a larger impact on catalysis for *E*. *coli* RPR compared to *P*. *furiosus* RPR. Finally, we provide data indicating that the absence of the S-domain and P18, or the P8/ P18-interaction in full-length RPR influences the charge distribution near the cleavage site in the RPR-substrate complex to a small but reproducible extent.

## Introduction

Almost all tRNAs carry a phosphate at their 5' ends due to the action of the endoribonuclease RNase P. Bacterial RNase P consists of one protein (C5), and one RNA subunit [1]. The composition of archaeal and eukarayal RNase P is more complex where the sole RNA subunit binds several proteins [2, 3]. Available data suggest that the catalytic activity resides in the RNA irrespective of origin, and the RNA alone can cleave various substrates in the absence of protein at high ionic strength [2, 4–7]. However, recent data demonstrate the presence of a protein only RNase P activity (PRORP), for example in human mitochondria and in *Arabidopsis thaliana*, that possesses the capacity to cut precursor tRNAs at the same site as RNase P [8, 9].

On the basis of secondary structure RNase P RNA (RPR) can be divided into different types. Type A [ancestral type; exemplified by *Escherichia coli* (*Eco*) RPR] and type B (Bacillus type) are the two main types among the bacteria [10]. Type A also exists in Archaea, as exemplified by *Pyrococcus furiosus* RPR, *Pfu* RPR [6]. Other types such as M and T have also been identified in Archaea [11–13]. Irrespective of type, two major domains can be identified, the specificity (S-) and the catalytic (C-) domains albeit in type T RPR the S-domain is degenerated [12–16]. The S-domain provides the binding site (TBS; TSL-binding site) for the pre-tRNA T-stem/ loop region referred to as TSL and the interaction to the TSL/ TBS-interaction [16–19]. The catalytic activity is associated with the C-domain and with respect to bacterial RPR substantial activity is retained when the S-domain is deleted [20–23]. It has also been suggested that the catalytic activity of archaeal RPR is associated with the C-domain [6, 24]. Although, a *cis* construct composed of the *Methanocaldococcus jannaschii* (archaeal type M) C-domain and pre-tRNA is catalytic [11] no cleavage activity *in trans* without protein has yet been demonstrated for an archaeal RPR lacking the S-domain. For type A RPR two interactions play important roles, the intra-domain P6-interaction in the C-domain and the P8/ 18-interaction involved in connecting the S- and the C-domains [18, 25, 26]. However, *Pfu* RPR lacks P18 and consequently the P8/ 18-interaction is missing [6]. The P6- and P8/ 18-interactions are absent in type B RPR, however, the intra-domain contact L5.1/L15.1 may fulfill the role of P6 in type B [10, 27]. Of interest, in this context we note that type T RPRs have degenerated S-domains but modeling suggest that P6 is present while P18 is absent [12, 13].

Disruption of the P6- or P8/ P18-interactions has been reported to affect the catalytic performance of the *Eco* RPR and the type A *Thermus thermophiles* RPR, *Tth* RPR [18, 28–30]. Compared to the full-size type A *Eco* RPR the cleavage efficiency of a model hairpin loop substrate by *Eco* CP RPR_wt_ is reduced almost 500-fold ([23]; see also Refs [20, 22]) while deleting the S-domain in the type B *Bacillus subtilis* (*Bsu*) reduces the catalytic performance several thousand-fold compared to full-size *Bsu* RPR [21]. Within type A RPR the C-domain residues in the P15-17 region interact with the 3' end of the precursor substrate and from the crystal structure it can be inferred that their structural position is influenced by the P6-interaction in full-size RPR [18]. Removing the P15-17 region in full-size *Eco* RPR_wt_, which also disrupts the P6-interaction, results in almost a 50000-fold reduction in catalytic efficiency [9]. For *Eco* CP RPR_wt_ no activity could be detected upon deleting P15-17 [22]. Inspection of the *Eco* RPR_wt_ structure reveals the possibility that residues involved in formation of P6 in full-size RPR can interact and resulting in an interaction mimicking P6 in *Eco* CP RPR_wt_, for convenience referred to as the "P6-mimic". Hence, we were interested in understanding whether the "P6-mimic" is formed and if so does it contribute to *Eco* CP RPR mediated catalysis. Moreover, deleting the S-domain results in disruption of the P8/ P18-interaction. This enabled us to assess the contribution (if any) of P18 to catalysis in an *Eco* CP RPR context and thereby get insights to its role during catalysis also for full-size RPR. This is of specific interest since the type A *Pfu* RPR_wt_ lacks P18 and show lower activity than *Eco* RPR_wt_ in the RNA alone reaction [6, 31]; see also Refs [24, 32]. We were therefor also interested in whether a *Pfu* RPR lacking the S-domain retains its catalytic activity even in the absence of protein.

Our data indicate that a "P6-mimic" is likely to be present in *Eco* CP RPR_wt_ and that its disruption reduce the cleavage efficiency. We also provide data suggesting that P18 does not influence the catalytic performance of *Eco* CP RPR_wt_ in cleavage of all ribo model hairpin loop substrates when the C5 protein is absent, which is not the case for full-size type A RPR. However, in the presence of the C5 protein a modest reduction in cleavage activity for *Eco* CP RPR_wt_ was detected upon deleting P18. Our data also show that deletion of the S-domain of *Pfu* RPR resulted in an RPR that is catalytically active in the absence of proteins. We also found that deletion as well as disruption of the P8/ P18-interaction in full-size *Eco* RPR lowers the cleavage efficiency of a model substrate. On the basis of our data combined with the fact that the P8/ P18-interaction (or P6) is not in direct vicinity of where substrate cleavage occurs we raise the possibility that the P8/ P18-interaction acts as a structural mediator between the TSL/ TBS-interaction site and the active center leading to positioning of chemical groups and Mg^2+^ that ensures correct and efficient cleavage.

## Materials and methods

### Preparation of substrates and RPR

The substrates were purchased from Dharmacon, USA and were purified on a 15% (w/v) denaturing PAGE gel followed by an overnight Bio-Trap extraction (Schleicher and Schuell, GmbH, Germany; Elutrap in USA and Canada) and phenol-chloroform extraction. γ-ATP 5' end-labeled substrates were generated and gel-purified using standard protocols.

The genes encoding full-size *Eco* RPR_wt_ (M1 RNA), *Eco* CP RPR_wt_ and *Pfu* RPR_wt_ have previously been described [22, 31, 33]. The genes encoding the variants *Eco* RPR_P18CUUG_, *Eco* RPR_G235_, *Eco* CP RPR_C83C84_, *Eco* CP RPR_G278G279_, *Eco* CP RPR_C83C84/G278G279_, *Eco* CP RPR_delP18_, *Eco* CP RPR_delP18P3Mini_, *Eco* CP RPR_31_, *Eco* CP RPR_31delP18_ and *Pfu* CP RPR_wt_ behind the T7 promoter were generated following the same procedure as outlined elsewhere [22, 31, 33] using the *Eco* CP RPR_wt_ and *Pfu* CP RPR_wt_ genes as template and appropriate oligonucleotides. *Eco* RPR_delP18_ was generated by replacing the 3' half of *Eco* RPR_wt_ with the 3' half of *Eco* CP RPR_delP18_ using appropriate restriction enzymes. The different RPRs were generated as run-off transcripts using T7 DNA-dependent RNA polymerase and PCR-amplified templates [34, 35]. The C5 protein was purified as described in [34, 36].

### Assay conditions

The cleavage reactions without the C5 protein were conducted in buffer C [50 mM 4-morpholineethanesulfonic acid (MES) and 0.8 M NH_4_Cl (pH 6.1)] at 37°C and 800 mM Mg(OAc)_2_ or as otherwise indicated (see Supporting information S1 Fig). The RPRs were pre-incubated at 37°C in buffer C and 800 mM Mg(OAc)_2_ for at least 10 min to allow proper folding before mixing with pre-heated (37°C) substrate. In all the experiments the concentrations of substrates were ≤0.02 μM while the concentrations of the different RPR variants were as indicated in Table and Figure legends.

Reactions with the C5 protein were done in buffer A [50 mM Tris-HCl (final pH 7.2), 5% (w/v) PEG 6000, 100 mM NH_4_Cl] and 10 mM Mg(OAc)_2_ as described in [34].

In the RPR alone reactions the k_app_ values were determined from experiments done under single turnover conditions where we measured the percentage of cleavage as a function of time (with C5 the RPR concentration varied between 0.004 and 0.009 μM). For the calculations we used the 5' cleavage fragment. To be able to compare with our previously reported data we refer to the so obtained rates as k_app_ values. The concentration of substrate was ≤0.02 μM while the RPR concentration varied dependent on RPR variant (see Table 1).

**Table 1:** Rate of cleavage (k_app_) of pATSerUG for RPR variants with and without the C5 protein as indicated.

RPR variant	Structural consequence	without C5	with C5
*Eco* RPR_wt_		12±0.5	2161±223
*Eco* RPR_P18CUUG_	P8/P18 disrupted G_314_CGA_317_ changed to C_314_UUG_317_	0.58±0.075	ND
*Eco* RPR_delP18_	P18 deleted	0.018±0.0039	ND
*Eco* CP RPR_wt_	S-domain removed	0.095±0.002	18±1.2
*Eco* CP RPR_C83C84_	"P6 mimic" disrupted	0.012±0.0006	ND
*Eco* CP RPR_G277G278_	"P6 mimic" disrupted	0.0034±0.0004	ND
*Eco* CP RPR _C83C84/G277G278_	"P6 mimic" restored	0.09±0.004	ND
*Eco* CP RPR_delP18_	P18 deleted	0.19±0.001	5±0.06
*Eco* CP RPR_delP18P3Mini_	P18 deleted P3 size reduced	0.1±0.006	3.6±0.3
*Eco* CP RPR_31_	"P6 mimic" deleted	0.002±0.00003	ND
*Eco* CP RPR_31delP18_	"P6 mimic" deleted P18 deleted	0.0024±0.00003	ND
*Pfu* RPR_wt_		0.040±0.015	ND
*Pfu* CP RPR_wt_	S-domain deleted	0.0047±0.0011	ND

Expressed as % of cleavage per min per pmol of RPR. The values are averages of at least three independent time-course experiments ± the maximum deviation from the average value; for *Eco* RPR_wt_ the value is based on two independent experiments. The concentrations of RPR (without C5) varied between 0.8 and 11 μM dependent on RPR variant while with C5 the concentration varied between 0.004 and 0.009 μM. The substrate concentration was ≤0.02 μM. ND = not determined.

Cleavage of pATSerU_am_G at 37°C was performed in buffer C, 0.8 M NH_4_Cl and 800 mM Mg(OAc)_2_ at pH 5.2, pH 6.1 and pH 7.2 [37, 38].

The cleavage reactions were terminated by adding double volumes of stop solution (10 M urea, 100 mM EDTA) and the products were separated on 25% (w/v) denaturing polyacrylamide gels.

### Structural probing

Structural probing of the *Eco* RPR variants, labeled at the 3'-end with [^32^P]pCp, was conducted using Pb^2+^-induced cleavage and limited RNase T1 digestion under native conditions as described elsewhere [34, 39, 40, 41]. Approximately 2 pmols of labeled RPR in 10 μl was pre-incubated for 10 min at 37°C in 50 mM Tris-HCl (pH 7.5), 100 mM NH_4_Cl and 10 mM MgCl_2_ together with 4 μM unlabeled tRNA. Cleavage was initiated by adding freshly prepared Pb(OAc)_2_ to a final concentration of 0.5 mM (or as indicated in Fig 4 legend) and the reaction was stopped after 10 min. In the digestion with RNase T1, the RPR was pre-incubated as described above. One unit RNase T1 was added followed by incubation on ice for 10 min. The reactions were stopped after 10 min by adding two volumes of stop solution (see above) and the products were analyzed on an 8% (w/v) denaturing polyacrylamide gel.

### RNase H cleavage

Approximately one μg of 3'-[^32^P]pCp labeled RPR was re-suspended in H_2_O and incubated for 3 min at 95°C. Following this the RPR was re-natured prior to the reaction at 55°C for 5 min in the buffer supplied by the company (20 mM Tris-HCl, 20 mM KCl, 10 mM MgCl_2_, 0.1 mM EDTA, 0.1 mM DTT and final pH 7.5; ThermoFisher Scientific) followed by incubation at room temperature. The RPR was mixed with 120 pmols of DNA oligonucleotides 1 (5'TGCCCT) or 2 (5'TGGGCT) and incubated in reaction buffer (see above) for 15 min at 28°C (similar results were obtained using 12 pmols of DNA oligonucleotides 1 or 2). The reaction was initiated by adding one unit RNase H (ThermoFisher Scientific) and the reaction was terminated after 30 min by adding double volumes of stop solution (see above). The reaction products were separated on 10% (w/v) denaturing polyacrylamide gels (see also Fig 3 legend and Ref [39]).

### Determination of k_app_ and the kinetic constants k_obs_, k_obs_/K^sto^ and K^sto^

The rate constants k_obs_ and k_obs_/K^sto^ were determined under saturating single-turnover conditions at pH 6.1 (where cleavage is suggested to be rate limiting) and 800 mM Mg^2+^ as described elsewhere [19, 23, 34].

**Fig 1:**
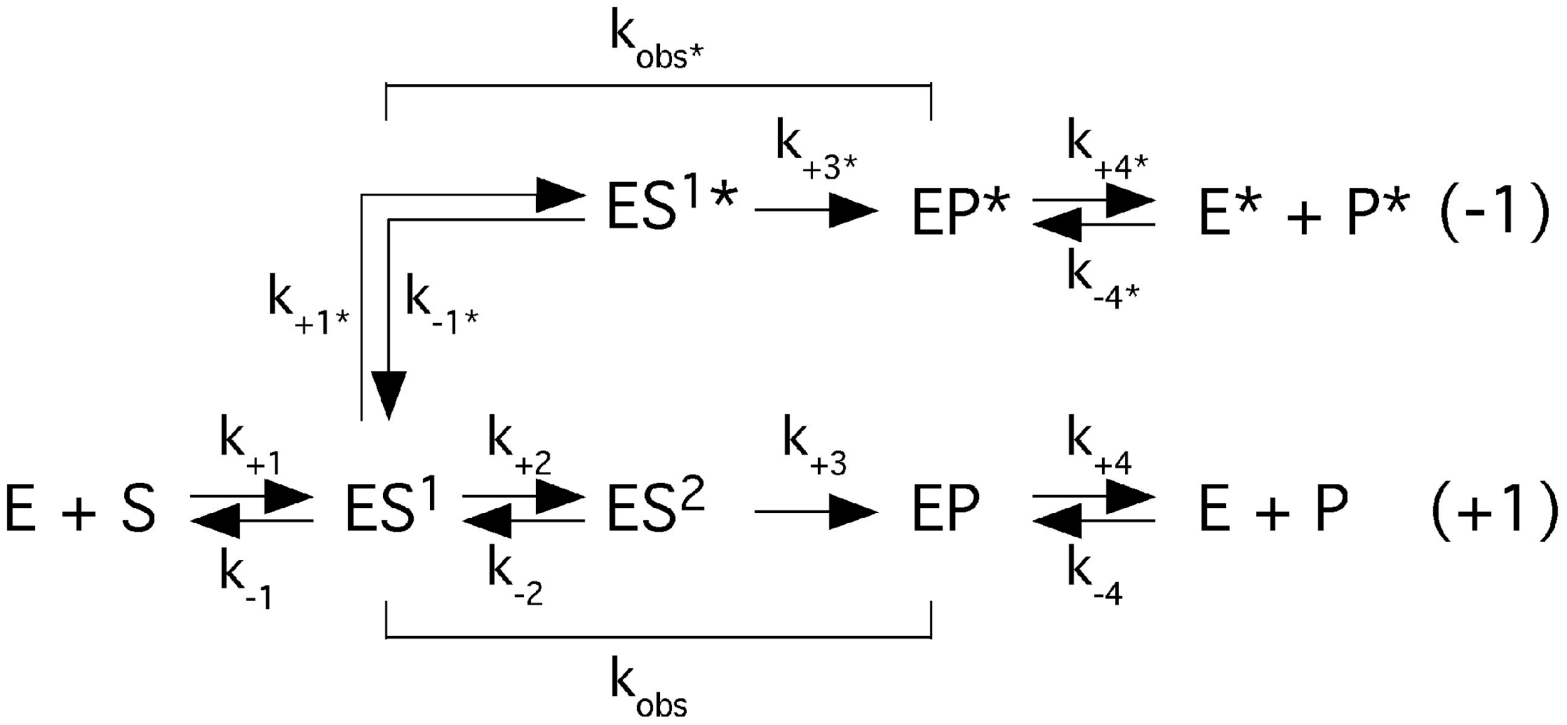
Simplified reaction scheme.

On the basis of the simplified scheme k_obs_ reflects the rate of cleavage (Fig 1). We have argued elsewhere that K^sto^ ≈ K_d_ in the *Eco* RPR-alone reaction [19, 23, 31, 34, 42, 43]. The final concentrations of the different RPR variants were between 0.4 and 47 μM (depending on the combinations of substrate and RPRs); the concentration of the pATSerUG substrate was 0.02 μM. To ensure that the experiments were done under the single-turnover conditions the lowest concentration of RPR was >10 times higher than the concentration of the substrate. For the calculations we used the 5' cleavage fragment and the time of cleavage was adjusted to ensure that the velocity measurements were in the linear range (*i*.*e*., ≤40% of the substrate had been consumed). To be able to compare with our previously published data k_obs_ and k_obs_/k^sto^ were obtained by linear regression from Eadie-Hofstee plots as described elsewhere [19, 23, 31, 34, 44, 45]. Each value was an average of at least three independent experiments and is given as a mean ± the deviation of this value.

## Results

### Structural probing of *Eco* CP RPR and full-size *Eco* RPR variants

#### Presence of a "P6-mimic" in *Eco* CP RPR_wt_

**Fig 2:**
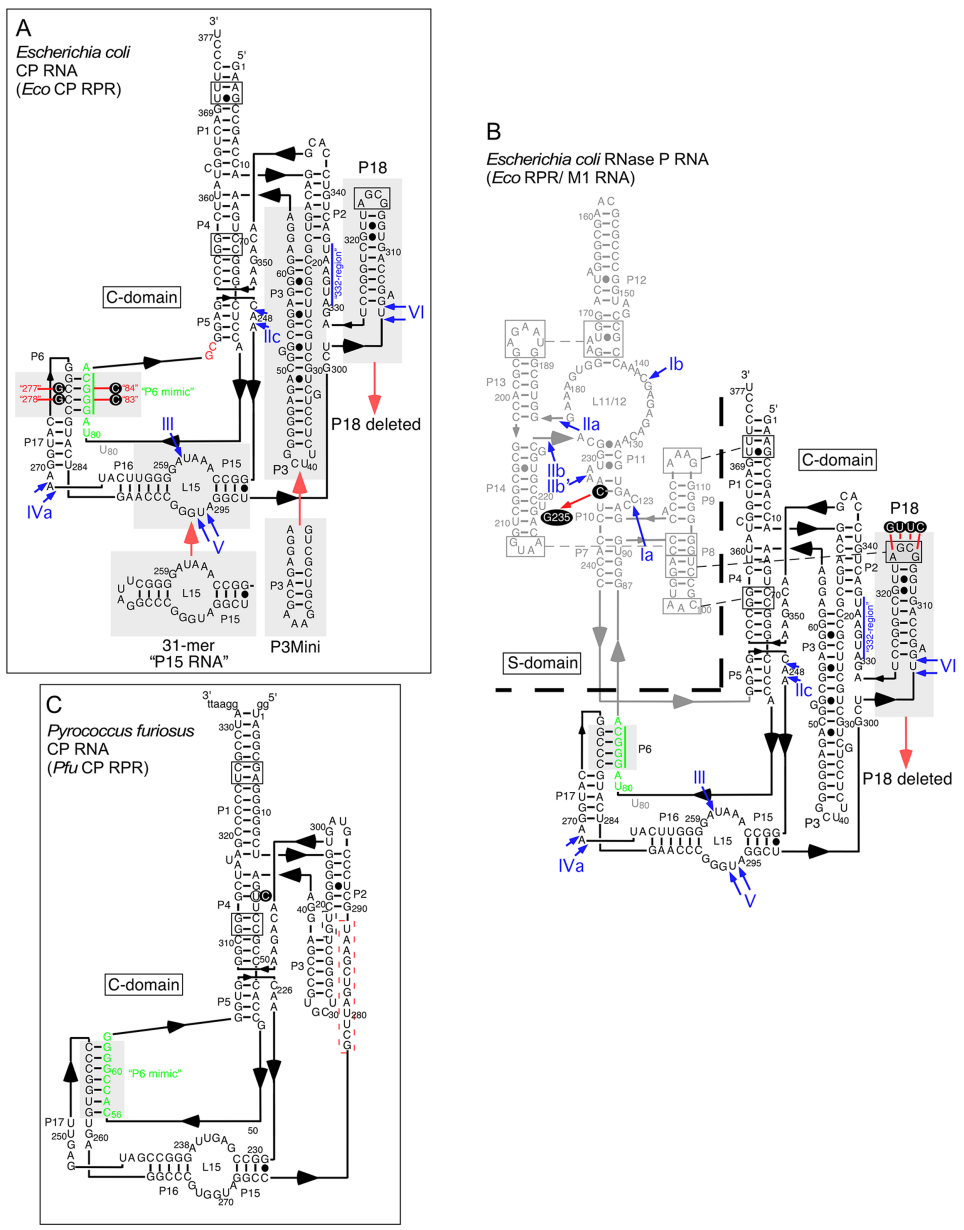
Predicted secondary structures of the type A RPRs as indicated. A. Type A *Eco* CP RPR [6, 46, 47], residues in green refer to residues involved in formation of the "P6-mimic" and residues highlighted (black circles) were changed as indicated: i) *Eco* CP RPR_C83C84_, *Eco* CP RPR_G277G278_ and *Eco* CP RPR_C83C84/G277G278_,. The G and C residues marked in red were added during the construction of the original *Eco* CP RPR_wt_ construct (see Refs [22, 23]). Replacements marked with grey boxes and red arrows indicate: i) the 31-mer "P15 RNA" module replacement of P15-17 in *Eco* CP RPR_31_ and *Eco* CP RPR_31delP18_), ii) P3Mini module replacement of the native P3 in *Eco* CP RPR_delP18P3Mini_ and iii) deletion of P18 in *Eco* CP RPR_delP18_, *Eco* CP RPR_31delP18_ and *Eco* CP RPR_delP18P3Mini_. The roman numerals and arrows in blue mark the Pb^2+^-induced cleavage sites IIc to VI (in Fig 2B, I to VI). For convenience the "332-region" is also indicated in blue and a vertical blue line. For details see the text. B. Type A *Eco* RPR, the residues constituting the S-domain are shown in light gray letters and the dotted line demarcates the border separating the S- and the C-domain. Residues highlighted (black circles) were changed as indicated to generate *Eco* RPR_P18CUUG_ and *Eco* RPR_G235_ while the red arrow indicate deletion of P18 generating *Eco* RPR_delP18_. C. Type A *Pfu* RPR derived from *Pyrococcus furiosus*. On the basis of the results using *Eco* CP RPR residues in green refer to residues involved in formation of the "P6-mimic".

**Fig 3:**
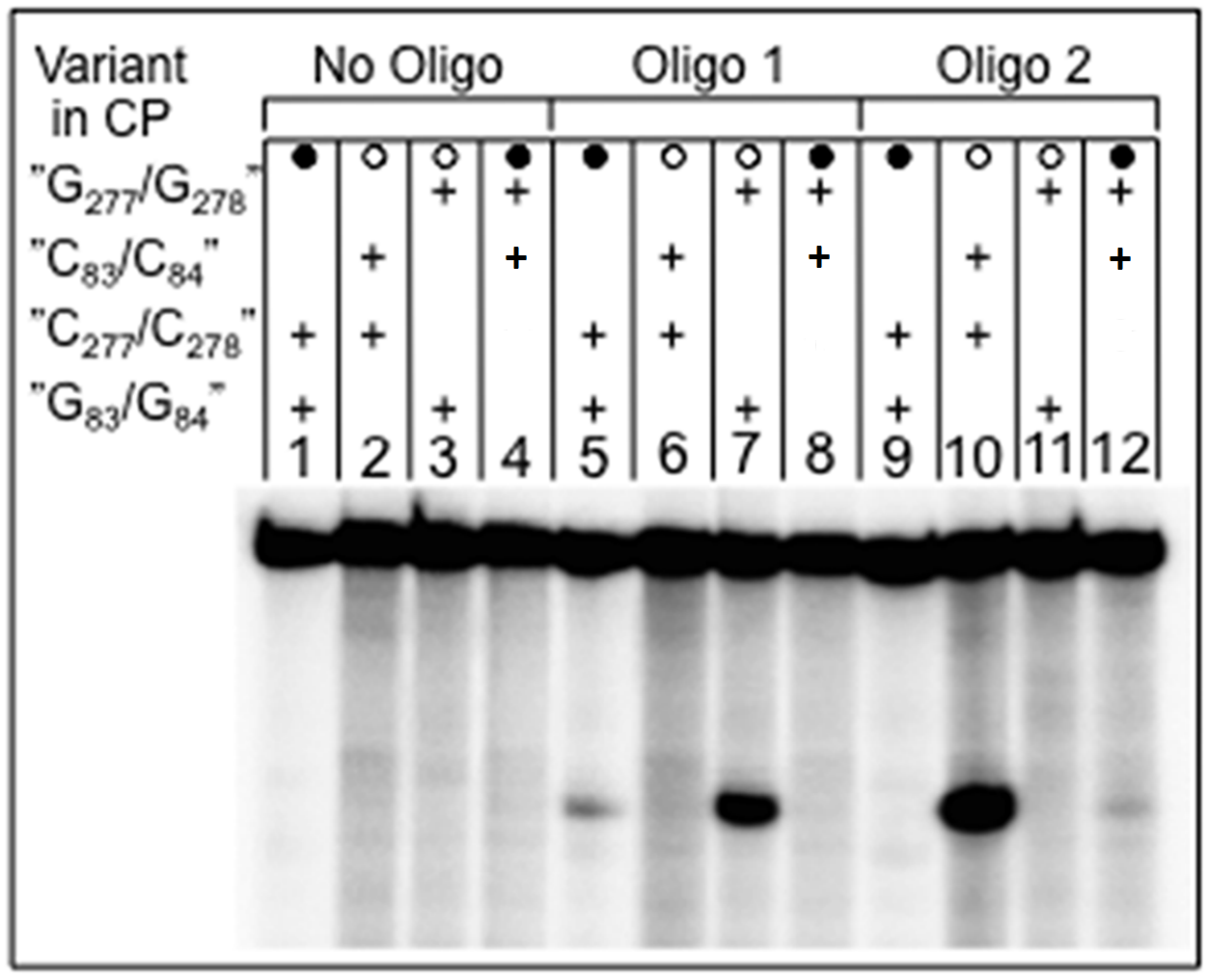
Formation of the "P6-mimic" in *Eco* CP RPR. Probing the accessibility of residues involved in formation of the "P6-mimic" in *Eco* CP RPR with RNase H and in the presence of DNA oligo 1 (5'TGCCCT, complimentary to residues (underlined) 5'A_81_GGGCA_86_ in *Eco* CP RPR_wt_ and *Eco* CP RPR_G277G278_) and DNA oligo 2 5'TGGGCT, complimentary to residues 5'A_81_GCCCA_86_ in *Eco* CP RPR_C83C84_ and *Eco* CP RPR_C83C84/G277G278_ as indicated. The black circles indicate when the "P6-mimic" can form while open circles refer to when it cannot form. For experimental and other details see text.

The "P6-mimic" might form in *Eco* CP RPR_wt_ since residues in the P17-loop that constitute one part of P6 are open to pair with other residues in *Eco* CP RPR as a result of deleting the S-domain (marked in green in Fig 2A). To test for the presence of the "P6-mimic" we generated the following *Eco* CP RPR variants (Fig 2A and Table 1): *Eco* CP RPR_C83C84_ ("P6-mimic" disrupted), *Eco* CP RPR_G277G278_ ("P6-mimic" disrupted), *Eco* CP RPR _C83C84/G277G278_ ("P6-mimic" restored), *Eco* CP RPR_31_ ("P6-mimic" disrupted due to replacement of the P15-17 domain with P15 RNA [22, 23]). These variants (except *Eco* CP RPR_31_) were probed with respect to the accessibility of residues 5'A_81_GGGCA_86_ (underlined residues altered in the respective CP RPR variant; Fig 2A) to RNase H in the presence of DNA oligonucleotides (i) 5'TGCCCT (oligo 1), complimentary to residues (underlined) 5'A_81_GGGCA_86_ in *Eco* CP RPR_wt_ and *Eco* CP RPR_G277G278_ and ii) 5'TGGGCT (oligo 2), complimentary to residues 5'A_81_GCCCA_86_ in *Eco* CP RPR_C83C84_ and *Eco* CP RPR_C83C84/G277G278_; Fig 2A; see also Ref [39]). We expected that disruption of the "P6-mimic" should result in RNase H cleavage of *Eco* CP RPR in the presence of oligo 1 and oligo 2 in a predictable manner. Subjection to cleavage with RNase H in the presence of either of the two DNA oligonucleotides indeed revealed strong cleavage for *Eco* CP RPR_G277G278_ (oligo 1), and *Eco* CP RPR_C83C84_ (oligo 2) as expected if the "P6-mimic" does not form. By contrast, for *Eco* CP RPR_wt_ and *Eco* CP RPR_C83C84/G277G278_ we did observe significant lower cleavage (Fig 3). We interpreted these data as an indication that the "P6-mimic" is likely to form in both *Eco* CP RPR_wt_ and *Eco* CP RPR_C83C84/G277G278_.

#### Structural impact of the P8/ P18-interaction and P18 on full-size *Eco* RPR and *Eco* CP RPR

To investigate the influence of P18 on the *Eco* CP RPR structure and its contribution to catalysis (see below) we generated the following variants (Fig 2A) *Eco* CP RPR_delP18_ (P18 deleted), *Eco* CP RPR_delP18P3Mini_ (P18 deleted, P3 size reduced) and *Eco* CP RPR_31delP18_ ("P6 mimic" and P18 deleted). For comparison we also generated two full-size variants, *Eco* RPR_delP18_ and *Eco* RPR_P18CUUG_, which both disrupt the P8/ P18-interaction albeit in different ways, in the former P18 is deleted while in the latter the P8/ P18-interaction is disrupted (Fig 2B). Comparing *Eco* CP RPR_wt_ and *Eco* RPR_wt_ allowed us also to assess whether removal of the S-domain (and the P8/ P18-interaction) affected the structure of the C-domain. First we studied the impact of the P8/ P18-interaction on full-size *Eco* RPR.

**Fig 4:**
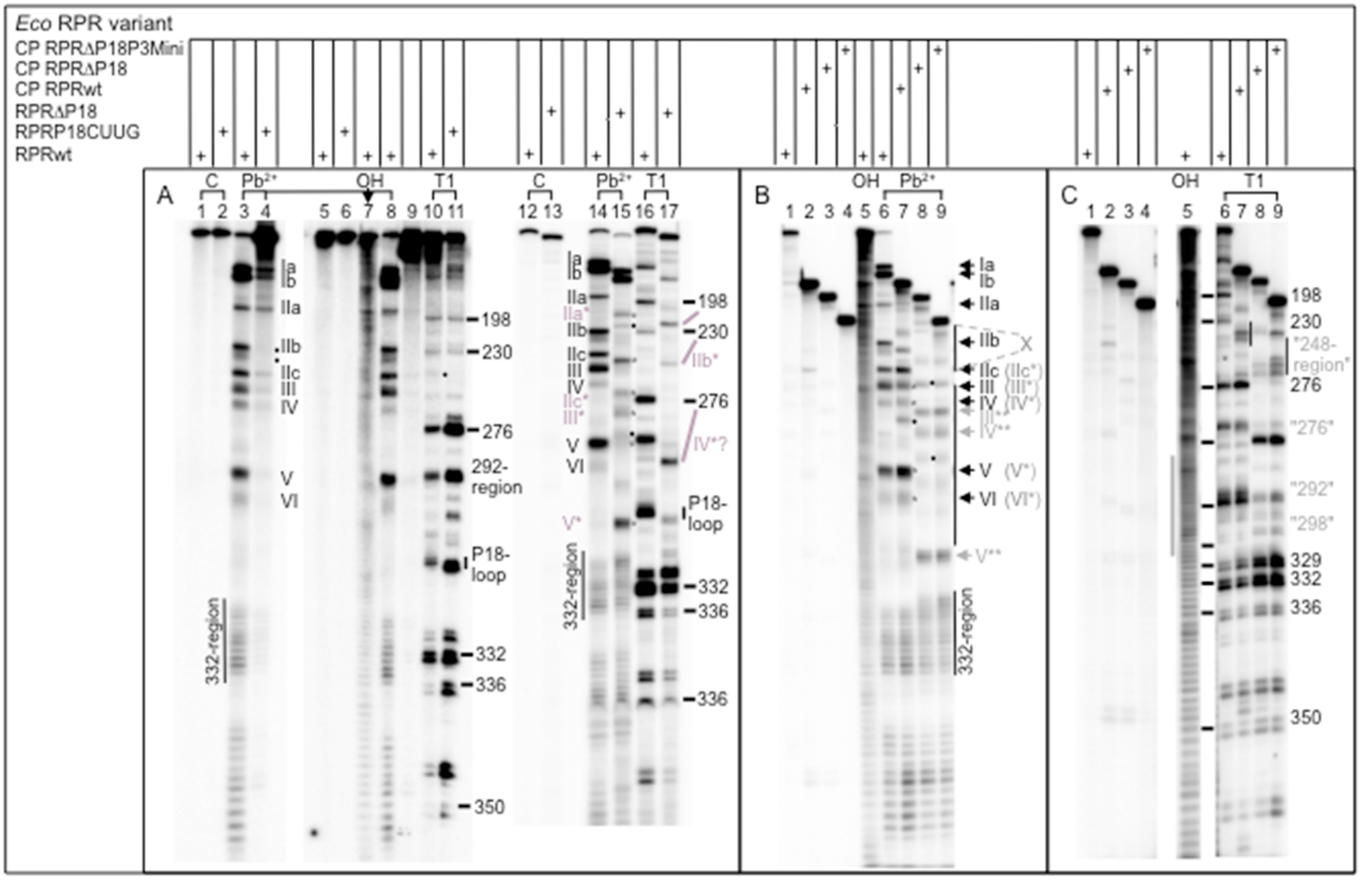
Structural probing of the *Eco* RPR variants with Pb^2+^ and RNase T1 as indicated. A. Pb^2+^-induced cleavage and RNase T1 digestion of *Eco* RPR_wt_ (Pb^2+^, lanes 3, 8 and 14; RNase T1, lanes 10 and 16), *Eco* RPR_P18CUUG_ (Pb^2+^, lane 4; RNase T1, lane 11) and *Eco* RPR_delP18_ (Pb^2+^, lane 15; RNase T1, lane 17). The Roman numerals mark the Pb^2+^-induced cleavage sites [41, 48] and the vertical line marks the cleavage sites in the 332-region [49]. Roman numerals in grey (IIa*, IIb*, IIc*, III* and V*; site IV* is questionable) mark the Pb^2+^ cleavage sites (see bands marked with grey *) and the grey lines the RNase T1 cleavage sites in *Eco* RPR_delP18_. The • mark differences comparing *Eco* RPR_wt_ and *Eco* RPR_P18CUUG_ or *Eco* RPR_delP18_. Incubations of RPRs without the addition of Pb^2+^ or RNase T1 lanes 1, 2, 5, 6, 12 and 13, and OH refer to the alkaline ladder (lane 7). The reactions were performed as outlined in Materials and Methods using 0.5 mM Pb(OAc)_2_ for *Eco* RPR_wt_ and *Eco* RPR_delP18_ while for *Eco* RPR_P18CUUG_ we used 10 mM Pb(OAc)_2_. B. Pb^2+^-induced cleavage of *Eco* RPR_wt_ (lane 6), *Eco* CP RPR_wt_ (lane 7), *Eco* CP RPR_delP18_ (lane 8) and *Eco* CP RPR_delP18P3Mini_ (lane 9). Roman numerals marked in black refers to the Pb^2+^-induced cleavage sites that are present in *Eco* RPR_wt_ and *Eco* CP RPR_wt_ while those marked in grey (*Eco* CP RPR_delP18_ and *Eco* CP RPR_delP18P3Mini_, IIc*, III* and V* likely correspond to the sites IIc, III and V present in *Eco* RPR_wt_ and *Eco* CP RPR_wt_ on the basis of the migration of the bands). Band marked with a grey * present in *Eco* CP RPR_delP18_ and *Eco* CP RPR_delP18P3Mini_ (X*) likely correspond to cleavage in the vicinity of residues that are part of the "P6-mimic". Bands marked with • refer to the appearance of new cleavage sites in *Eco* CP RPR_wt_ that are not detected in the full-size *Eco* RPR_wt_. Lanes 1–4 incubations of RPRs in the absence of Pb^2+^ and OH = alkaline ladder (lane 5). For experimental details see Materials and methods. C. RNase T1 cleavage of *Eco* RPR_wt_ (lane 6), *Eco* CP RPR_wt_ (lane 7), *Eco* CP RPR_delP18_ (lane 8) and *Eco* CP RPR_delP18P3Mini_ (lane 9) as indicated. Lanes 1–4 incubations of the RPRs without the addition of RNase T1 while OH = alkaline ladder (lane 5). The grey vertical line mark the region that constitute P18 in *Eco* RPR_wt_ and *Eco* CP RPR_wt_ whereas the numbers given in grey mark the RNase T1 cleavage sites in *Eco* CP RPR_delP18_ and *Eco* CP RPR_delP18P3Mini_ and these sites likely correspond to the sites detected in *Eco* RPR_wt_ and *Eco* CP RPR_wt_, *e*.*g*. the "276" (marked in grey) cleavage site correspond to the cleavage site at 276 (marked in black). The vertical black lines mark the "248-region" in the CP RPRs.

Structural probing of *Eco* RPR_wt_ and *Eco* RPR_P18CUUG_ with Pb^2+^ and RNase T1 (Fig 4A) suggested that disruption of the P8/ P18-interaction affected the P18 structure and the region near Pb^2+^-induced cleavage sites IIb and possibly also IIb' (marked with a dot and absent in *Eco* RPR_P18CUUG_ at 10 mM Pb^2+^, cf. lanes 3 and 4; see also Fig 4A legend; Pb^2+^ cleavage sites are marked in Fig 2). We also noted that a weak RNase T1 cleavage product (marked with a dot; Fig 4A, cf. lanes 10 and 11) was absent just upstream of the Pb^2+^-induced cleavage site IIc at A248 in *Eco* RPR_P18CUUG_ (Fig 2B), where A248 is close to the tRNA 5' end in the RNase P-tRNA complex [18]. Of note, a higher concentration of Pb^2+^ was needed for *Eco* RPR_P18CUUG_ [10 mM *vs*. 0.5 mM for *Eco* RPR_wt_; see Fig 4A, cf. lanes 3 (or 8) and 4], which might indicate an effect on Pb^2+^ binding affinities perhaps due to a more flexible structure. Deletion of P18 (*Eco* RPR_delP18_) also resulted in some changes in the Pb^2+^ cleavage pattern with the appearance of an extra band in the IIa/IIb region (marked with a dot, Fig 4, cf. lanes 14 and 15). Also, Pb^2+^ mediated cleavage at IIb in *Eco* RPR_delP18_, which is in contrast compared to *Eco* RPR_P18CUUG_ (Fig 4A, cf. lanes 4 and 15). Moreover, apart from the P18 region the RNase T1 cleavage patterns were similar comparing *Eco* RPR_wt_ and *Eco* RPR_delP18_ (Fig 4A, cf. lanes 16 and 17). Together these data indicate some influence on the overall RPR structure, apart from the P18 region, when the P8/ P18-interaction is absent (or disrupted), in particular in the region near the Pb^2+^ cleavage site IIb.

Next we investigated the influence of P18 on the structure of *Eco* CP RPR_wt_, which lacks the S-domain (and the P8/ P18-interaction). The data are shown in Fig 4B (cleavage with Pb^2+^) and Fig 4C (cleavage with RNase T1). As expected, gel mobility of equivalent cleavage products (relative to full-size *Eco* RPR_wt_) of *Eco* CP RPRs lacking P18, *Eco* CP RPR_delP18_ and *Eco* CP RPR_delP18P3Mini_, shifted (cf. shift of bands comparing the patterns for *Eco* RPR_wt_ and *Eco* CP RPR_wt_, *e*.*g*. the band that corresponds to Pb^2+^-induced cleavage site V; Fig 4B, compare lanes 6 and 7 with lanes 8 and 9). However, the Pb^2+^-induced cleavage sites IIc, III, and V as well as those near residue 332 are most likely still present in the *Eco* CP RPR variants (Fig 4B, cf. lanes 6 and 7 vs. 8 and 9; the grey * marks the shift of sites IIc, III and IV, and referred to as IIc*, III* and IV* in *Eco* CP RPR_delP18_ and *Eco* CP RPR_delP18P3Mini_). This suggested that the metal(II)-ion binding sites in the vicinity of these sites likely remain intact despite the absence of the S-domain nor do they depend on the presence of P18 or the length of P3. The Pb^2+^-induced cleavage at sites IIc, III and V, and in the 326-335-region are most likely also present in the *Eco* CP RPR constructs that lack P18 (Fig 4B, cf. lanes 8 and 9). Site VI that is present in both *Eco* RPR_wt_ and *Eco* CP RPR_wt_ is absent in the *Eco* CP RPR variants that lack P18 (see also Ref [49]). The bands upstream of IIc (marked with X; Fig 4B) might possibly be the result of Pb^2+^-induced cleavage near residues constituting the "P6-mimic". RNase T1 cleavage of the *Eco* CP RPR variants revealed that most of the cleavage sites detected using full-size *Eco* RPR_wt_ were present (Fig 4C, cf. lanes 6–9). However, we noted one apparent difference in the region referred to as the "248-region" (marked with a short vertical grey line, Fig 4C, cf. lanes 8 and 9) where we detected new and stronger cleavage.

Taken together, the P8/ P18-interaction appears to affect the overall structure of full-size RPR to a certain degree (*e*.*g*., cf. Fig 4A lanes 3 and 4) while deletion of the S-domain and P18 does not affect the overall structure of the C-domain to any significant extent except for changes, in particular in the P18-region (as expected) and the region referred to as the "248-region". From our data it also appears that the overall structure of the C-domain is not much affected by deleting the S-domain (compare *Eco* RPR_wt_ and *Eco* CP RPR_wt_).

### Impact of the "P6-mimic", P18 and P8/ P18-interaction on the catalytic performance of *Eco* CP RPR and full-size *Eco* RPR

**Fig 5:**
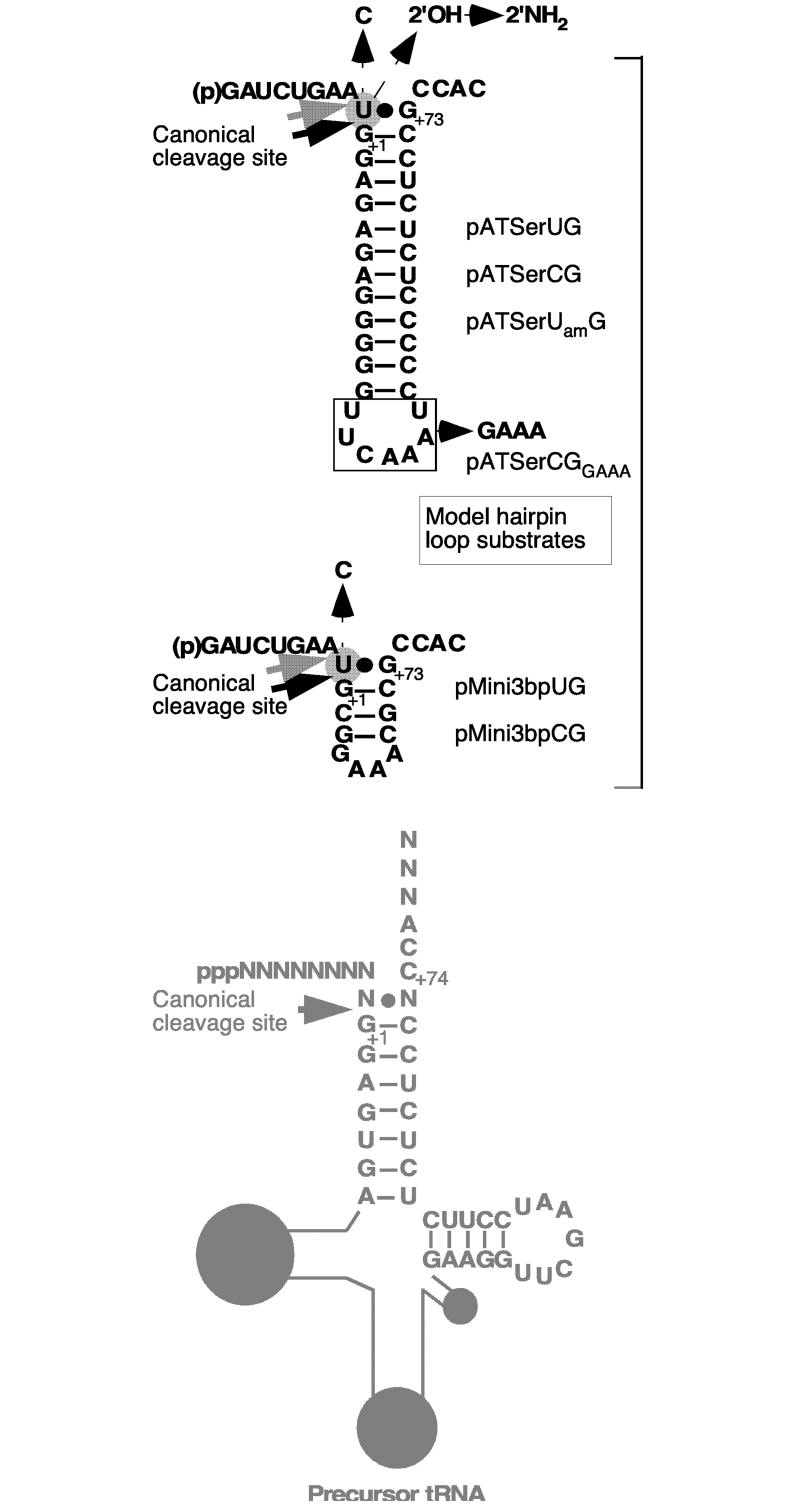
Secondary structures of the model hairpin loop substrates used in this study. The arrows mark the cleavage sites as indicated: black arrows mark the canonical cleavage sites between residues -1 and +1, and gray arrows mark the alternative site between residues -2 and -1. The differences with respect to the identity of residue -1 are indicated, as is the replacement of the 2'OH to 2'NH_2_ at the -1 position in pATSerUG. The numbering in the vicinity of the cleavage sites corresponds to that used for tRNA and precursor tRNA [51]. The precursor tRNA (pre-tRNA) is included to illustrate the design of the model hairpin loop substrates and their structural differences relative to full-length pre-tRNA substrates (the grey circles correspond to the D-loop, anticodon and variable-loop).

As substrates we used different well-characterized model hairpin-loop substrates, which are derived from the *E*. *coli* tRNA^Ser^Su1 precursor (Fig 5; [34, 39, 50], and references therein). The longer substrates, pATSerUG and pATSerCG, can interact with the TBS-site in the RPR while the shorter, pMini3bpUG and pMini3bpCG, cannot. As we reported elsewhere optimal cleavage of these substrates by *Eco* RPR_wt_ or *Eco* CP RPR_wt_ requires higher Mg^2+^-concentrations [16, 19, 23, 37]. This is also the case for cleavage of pATSerUG with *Eco* CP RPR_delP18_ and *Eco* CP RPR_delP18P3Mini_ (variants described below) where optimal cleavage of pATSerUG was reached at approximately 800 mM Mg^2+^ (Supporting Information S1 Fig, we assume this to be the case irrespective of which RPR substrate combination used in this study). On the basis of these data and our earlier studies, the experiments presented here were done at 800 mM Mg^2+^. The choice of this Mg^2+^ concentration also allowed us to directly compare the results with our previously published data. Cleavage in the presence of the C5 protein was done at 10 mM Mg^2+^ ([4, 33] and Materials and methods). Finally, *Eco* RPR_wt_ and *Eco* CP RPR_wt_ can cleave model hairpin loop substrates at the correct (or canonical) site between residue -1 and +1 (referred to as the +1 site) and at the alternative site between positions -1 and -2 (referred to as miscleavage or the -1 site; Fig 5; see *e*.*g*., Refs [16, 19, 34, 50]).

#### The "P6-mimic" affects the catalytic performance of Eco CP RPR_wt_

**Fig 6:**
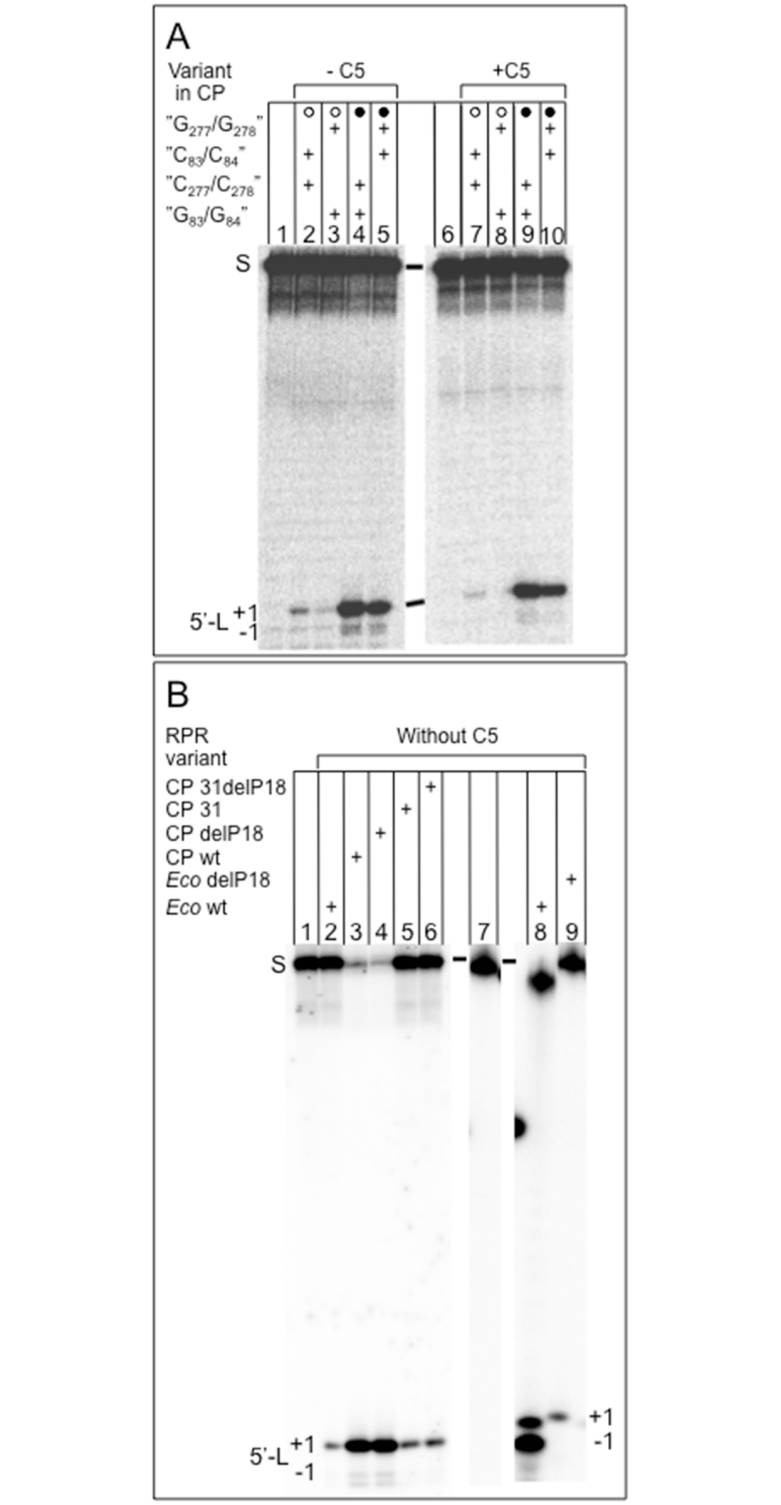
Cleavage of the model hairpin loop substrate pATSerUG. A. Cleavage with different *Eco* CP RPR carrying changes that affect the "P6-mimic" (Fig 2A). The experiments were done with and without the C5 protein as indicated. Reactions without the C5 protein were performed in buffer C and 800 mM Mg^2+^ (cf. lanes 1 to 5) while those with the protein were done in buffer A and 10 mM Mg^2+^ (cf. lanes 6 to 10). All the reactions were done at 37°C and black and open circles as defined above. The concentrations of the RPRs were 0.7 nM with C5 and 2.7 μM without. The concentration of the C5 protein was 0.76 μM (empirically determined) and ≤0.02 μM for pATSerUG. Incubations were 30 min in all cases. Lane 1, pATSerUG alone; lane 2, *Eco* CP RPR_C83C84_; lane 3, *Eco* CP RPR_G277G278_; lane 4, *Eco* RPR_wt_; lane 5, *Eco* CP RPR_C83C84/G277G278_; lane 6, pATSerUG alone; lane 7, *Eco* CP RPR_C83C84_; lane 8, *Eco* CP RPR_G277G278_; lane 9, *Eco* RPR_wt_; lane 10, *Eco* CP RPR_C83C84/G277G278_. S = pATSerUG, 5'-L = 5' cleavage fragments and +1 and -1 marks were cleavage had occurred (see Fig 5 and text for details). B. Cleavage with various *Eco* RPR and CP RPR variants as indicated. The concentration of pATSerUG was ≤0.02 μM while the concentrations of the RPR varied: *Eco* CP RPR_wt_ 8.2 μM (lane 3), *Eco* CP RPR_delP18_ 9.2 μM (lane 4), *Eco* CP RPR_31_ 9.0 μM (lane 5), *Eco* CP RPR_31delP18_ 10.2 μM (lane 6), and *Eco* RPR_delP18_ 3.2 μM (lane 9). The reaction times were 20 min for *Eco* CP RPR_wt_ and *Eco* CP RPR_delP18_, 60 min for *Eco* CP RPR_31_, *Eco* CP RPR_31delP18_ and *Eco* RPR_delP18_. Controls, incubation of pATSerUG alone without RPR (lane 1), cleavage of pATSerUG (lane 2) and pATSerCG_GAAA_ (known to cleave at +1 and -1, see 18 Wu et al. 2011; lane 8) with *Eco* RPR_wt_. S = substrate, 5'-L = 5' cleavage fragments and +1 and -1 marks cleavage sites (see text for details).

As shown in Fig 6A both *Eco* CP RPR_C83C84_ and *Eco* CP RPR_G277G278_ cleaved pATSerUG with reduced efficiency compared to *Eco* CP RPR_wt_ and *Eco* CP RPR_C83C84/G277G278_. This was the case in particular in the RNA alone reaction. Determination of the rate of cleavage (k_app_; Table 1) for these *Eco* CP RPR variants without C5 corroborated these findings with 8- and 28-fold lower k_app_ values for *Eco* CP RPR_C83C84_ and *Eco* CP RPR_G277G278_, respectively, compared to *Eco* CP RPR_wt_. For *Eco* CP RPR_31_, which cannot form the "P6-mimic" (Fig 2A; substitution of P15-17 with P15 RNA) we detected an almost 50-fold reduction in k_app_ (Table 1; see also Fig 6B and below). The higher impact in response to replacing the P15-17 domain with P15 RNA might reflect a structural effect on establishing the pairing between the 3'ACC in the substrate and the RPR [51].

Together with the structural probing data discussed above suggested that the "P6-mimic" is likely to be present in *Eco* CP RPR_wt_ and that it contributes to its catalytic performance.

#### The P8/ P18-interaction influences cleavage of pATSerUG by full-size Eco RPR

Before analyzing the impact of P18 on catalysis in the *Eco* CP RPR context we first inquired whether the P8/ P18-interaction influences cleavage of the model hairpin loop substrate pATSerUG in the full-size *Eco* RPR context. Hence, we used the *Eco* RPR_delP18_ and *Eco* RPR_P18CUUG_ variants (see above), which allowed us also to assess the response upon deleting P18 (*Eco* RPR_delP18_) and "disruption" of the P8/ P18-interaction (*Eco* RPR_P18CUUG_). Both these variants cleaved pATSerUG mainly at the +1 site with reduced efficiency (Fig 6B, cf. lane 9; and not shown). Compared to *Eco* RPR_wt_, the cleavage rates of pATSerUG (k_app_; Table 1) for *Eco* RPR_P18CUUG_ and *Eco* RPR_delP18_ were reduced ≈20-fold and almost 700-fold, respectively. Determination of the kinetic constants under single turnover conditions revealed that "disruption" of the P8/ P18-interaction resulted in a ≈20- and 160-fold decrease in k_obs_ and k_obs_/K^sto^, respectively, while deleting P18 lowered both k_obs_ (and k_obs_/K^sto^) >3000-fold (Table 2; cf. values for *Eco* RPR_wt_, *Eco* RPR_P18CUUG_ and *Eco* RPR_delP18_). The K^sto^ values correspond to ≈ K_d_ values (see Materials and methods) and no difference was detected comparing *Eco* RPR_wt_ and *Eco* RPR_delP18_ while for *Eco* RPR_P18CUUG_ K^sto^ was ≈10-fold higher. These data suggest that the P8/ P18-interaction influence cleavage of pATSerUG, which is consistent with previous findings using pre-tRNAs [28–30]. Also, while deleting P18 (*Eco* RPR_delP18_) affected k_obs_ "disruption" of the P8/ P18-interaction resulted in changes in both k_obs_ and K^sto^ (see Discussion).

**Table 2:** Kinetic constants for cleavage of pATSerUG with RPR variants at 800 mM Mg^2+^ as indicated.

RPR variant	k_obs_ (min^-1^)	k_obs_/K^sto^ (= k_cat_/K_m_) (min^-1^ x μM^-1^)	K^sto^ (≈K_d_) (μM)	ΔΔG# (kcal)
*Eco* RPR_wt_	12^1^^,^^a^	19^1^^,^^a^	0.63	1
*Eco* RPR_P18CUUG_	0.50±0.09^a^	0.12±0.01^a^	4.3	+3.1
*Eco* RPR_delP18_	0.0033±0.00018	0.006±0.0007	0.56	+5
*Eco* CP RPR_wt_	0.34^2^^,^^a^	0.04^2^^,^^a^	8.3	+3.8
*Eco* CP RPR_delP18_	0.32±0.01^a^	0.036±0.002^a^	8.9	+3.9
*Pfu* RPR_wt_	0.058^1^^,^^a^	0.03^1^^,^^a^	1.9	1
*Pfu* CP RPR_wt_	0.016±0.003^a^	0.0018±0.00035^a^	8.9	+1.7
*Tth* RPR_wt_ (without C5)	ND	25^3^^,^^b^	ND	1
*Tth* RPR_P18(304/27)_ (without C5)	ND	1.4^3^^,^^b^	ND	+1.8
*Eco* RPR_wt_ (without C5)	ND	2.2^4^^,^^c^	ND	1
*Eco* RPR_P18UUCG(L18m)_ (without C5)	ND	0.2^4^^,^^c^	ND	+1.5
*Eco* RPR_wt_ (with C5)	ND	568^4^^,^^c^	ND	1
*Eco* RPR_P18UUCG(L18m)_ (with C5)	ND	237^4^^,^^c^	ND	+0.54
*Eco* RPR_wt_ (without C5)	ND	0.012^5^^,^^d^	ND	1
*Eco* RPR_delP18_ (without C5)	ND	0.0023^5^^,^^d^	ND	+1.0

^#^ΔΔG values (change with respect to the RPR_wt_ in each case) were calculated using k_obs_/K^sto^ (k_cat_/K_m_) values and ΔΔG = -RTln(k_obs_/K^sto^)_mu_/(k_obs_/K^sto^)_wt_ [52]. The experiments were conducted under single-turnover conditions at 800 mM Mg^2+^ and pH 6.1 as described in Materials and Methods. The concentration of substrate was ≤0.02 μM while the concentration of the different RPR variants varied dependent on RPR and substrate as stated in Materials and Methods. Numbers are averages of at least three independent experiments ± the maximum deviation of the average value.

Substrates used in the different reports were:

^a^pATSerUG model hairpin loop substrate;

^b^pre-tRNA^Gly^ from *T*. *thermophilus*;

^c^pre-tRNA^Tyr^Su3 from *E*. *coli*;

^d^pre-tRNA^Asp^ from *B*. *subtilis*.

Values taken from:

^1^Sinapah *et al*. 2011 [31];

^2^Wu et al. 2012 [34];

^3^Schlegl *et al*. 1994 [28];

^4^Pomeranz-Krummel and Altman, 1999 [30];

^5^Haas *et al*. 1994 [29], values based on the experiment done at 1M NH_4_Cl, at 3M NH_4_Cl no difference in k_cat_/K_m_ indicating that lack of P18 can be compensated for by increasing the ionic strength.

#### P18 does not influence the catalytic performance for *Eco* CP RPR

On the basis of the data discussed above one prediction was that this might also be the case for *Eco* CP RPR (see above). However, given that P18 helps to connect the S- and C-domains [18, 46] another possibility was that P18 does not affect catalysis since in *Eco* CP RPR the S-domain is missing (Fig 2A). To test this, and get insight into the contribution of P18 to catalysis, we studied cleavage of pATSerUG, pATSerCG, pMini3bpUG and pMini3bpCG (Fig 5) followed by determinations of the rate constant k_app_ (for pATSerUG) without the C5 protein and for a selected few in its presence (Table 1). We also determined the kinetic constants k_obs_ and K^sto^ in the absence of C5 using pATSerUG as substrate (see Materials and methods; Table 2) and *Eco* CP RPR_wt_ and *Eco* CP RPR_delP18_ (P18 deleted). For *Eco* CP RPR_delP18P3Mini_ (P18 deleted, P3 size reduced) and *Eco* CP RPR_31delP18_ ("P6-mimic" and P18 deleted) we only determined k_app_ values (Table 1).

**Fig 7:**
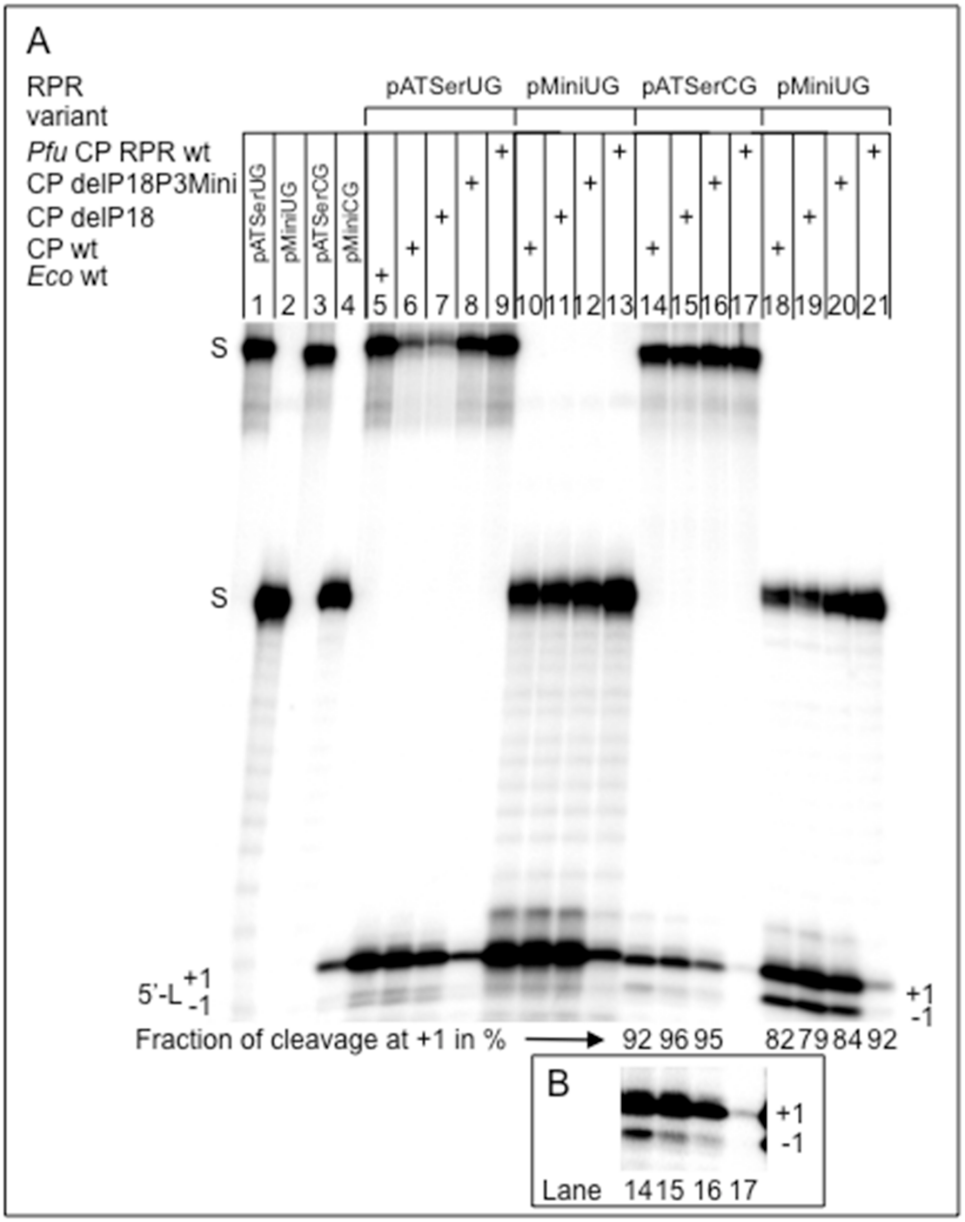
Cleavage of different model hairpin loop substrates as indicated. A. Cleavage of pATSerUG with *Eco* RPR_wt_ (0.8 μM; lane 5), *Eco* CP RPR_wt_ (20.5 μM; lanes 6, 10, 14 and 18), *Eco* CP RPR_delP18_ (23 μM; lanes 7, 11, 15 and 19), *Eco* CP RPR_delP18P3Mini_ (27 μM; lanes 8, 12, 16 and 20) and *Pfu* CP RPR_wt_ (20 μM; lanes 9, 13, 17 and 21). The concentration of substrates was ≤0.02 μM. Reaction times were 15 min (pATSerUG and pMini3bpUG) and 90 min (pATSerCG and pMini3bpCG) irrespective of RPR variant. In the case when full-size *Eco* RPR_wt_ was used the reaction time was 4 sec. The negative controls, incubation without RPR for 90 min (pATSerUG; lane 1), (pMini3bpUG; lane 2), (pATSerCG; lane 3) and (pMini3bpCG; lane 4). 5'-L marks the migration of the 5' cleavage fragments as a result of cleavage at +1 and at -1. Lanes 14–16 and 18–21, the numbers correspond to the frequency of cleavage at +1 expressed in percentage. The numbers are averages of at least three independent experiments with the following errors: 92±0.64 (lane 14), 96±0.07 (lane 15), 95±0.27 (lane 16), 82±1.4 (lane 18), 79±0.5 (lane 19), 84±0.65 (lane 20) and 92±0.5 (lane 21). For experimental details Materials and methods (see also Ref [50]). B. Lanes 14–17 overexposure of the 5' cleavage fragments shown in panel A.

The different *Eco* CP RPR variants with and without P18 cleaved the four model hairpin loop substrates preferentially at the correct position +1 (see Figs 6B and 7, cleavage with *Eco* CP RPR_wt_, *Eco* CP RPR_delP18_ and *Eco* CP RPR_delP18P3Mini_; for *Eco* CP RPR_31_ and *Eco* CP RPR_31delP18_ we only tested cleavage of pATSerUG, Fig 6B, cf. lanes 5 and 6; see above). Consistent with our previous data [16, 23, 34] the cleavage efficiencies for pATSerCG and pMini3bpCG were lower than using pATSerUG and pMini3bpUG (Fig 7; note that longer reaction times were needed to cleave pATSerCG and pMini3bpCG). Moreover, the cleavage efficiency of pATSerUG by *Eco* CP RPR_31delP18_ (and *Eco* CP RPR_31_; see above) was reduced compared to *Eco* CP RPR_wt_ (Fig 6B; Table 1, see below).

The substrates with C_-1_ (pATSerCG and pMini3bpCG; Fig 7, cf. lanes 14–17 and 18–21) were cleaved at the alternative site -1 irrespective of *Eco* CP RPR variant while the U_-1_ substrates were cleaved only with a low frequency at -1. A comparison of cleavage of pATSerCG and pMini3bpCG suggested that the latter is cleaved more frequently at -1 by the *Eco* CP RPR variants. The reason to this is at present unclear, however, it might be related to that these two substrates interact differently with *Eco* CP RPRs and/ or that the positioning of Mg^2+^ in the vicinity of the respective cleavage site differs. For example it has been suggested that residues near the conserved U69 in *Eco* RPR (Fig 2B) interact with the residue positioned five bases 3' of the cleavage site [53] and pMini3bp only has a stem of three base pairs (Fig 5).

Consistent with our previous data [23] *Eco* CP RPR_wt_ cleaved pATSerUG with a reduced rate (k_app_ decreased ≈100-fold) both with and without the C5 protein compared to full-size *Eco* RPR_wt_ (Table 1). In contrast to full-size *Eco* RPR, where "disruption" (*Eco* RPR_P18CUUG_) of the P8/ P18-interaction resulted in a 20-fold (or almost 700-fold upon deleting P18; see above) reduction in k_app_, deleting P18 in *Eco* CP RPR did not affect k_app_ (if anything, there was a modest ≈two-fold increase for *Eco* CP RPR_delP18_). In the presence of the C5 protein, there was a modest three- to four-fold decrease in k_app_ for the *Eco* CP RPR variant lacking P18 (Table 1; cf. values for *Eco* CP RPR_wt_, *Eco* CP RPR_delP18_ and *Eco* CP RPR_delP18P3Mini_).

Determination of the kinetic constants for cleavage of pATSerUG without C5 corroborated the data presented in Table 1 and revealed no change in either k_obs_ or K^sto^ when P18 in *Eco* CP RPR was deleted (Table 2; cf. values for *Eco* CP RPR_wt_ and *Eco* CP RPR_delP18_). These data are in contrast to full-size *Eco* RPR were deleting and "disrupting" the P8/ P18-interaction affected k_obs_ and k_obs_/K^sto^, respectively (see above; Table 2). Of note, the K^sto^ values (≈K_d_, see above) for *Eco* RPR_P18CUUG_ and *Eco* CP RPR_wt_ (or *Eco* CP RPR_delP18_) only differed by a factor of two (Table 2).

Taken together, these data emphasized the importance of the S-domain and the P8/ P18-interaction for catalysis and substrate binding for full-size *Eco* RPR while P18 does not contribute to the catalytic performance of *Eco* CP RPR to any significant extent. However, the presence of P18 in full-size *Eco* RPR that cannot properly interact with P8 does affect pATSerUG binding whereas its absence does not (see Discussion). Also, comparing k_app_ values (Table 1) for *Eco* CP RPR_delP18_ and *Eco* CP RPR_delP18P3Mini_ suggested that the length of P3 does not appear to influence the catalytic performance in an *Eco* CP RPR context.

#### The C-domain derived from *Pyrococcus furiosus* (*Pfu*) is catalytically active in the absence of the S-domain and protein

The type A *Pfu* RPR lacks P18 (Fig 2C and S2 Fig) and it is catalytic also in the absence of the S-domain but only in the presence of proteins ([6]; see Discussion). Full-size *Pfu* RPR_wt_ alone cleaves the model hairpin substrates used above at high Mg^2+^ concentration [31]. To test whether *Pfu* RPR_wt_ is catalytically active also without the S-domain and protein we generated *Pfu* CP RPR_wt_ (Fig 2C). Indeed, *Pfu* CP RPR_wt_ cleaved pATSerUG, pATSerCG, pMini3bpUG and pMini3bpCG mainly at the correct position +1 (Fig 7, cf. lanes 9, 13, 17 and 21). In addition, *Pfu* CP RPR_wt_ cleaved pMini3bpCG at the alternative site -1 (Fig 7, lane 21) while we could not detect any cleavage of pATSerCG at -1 (Fig 7B, lane 17). However, this could be because *Pfu* CP RPR_wt_ cleaved pATSerCG with a very low efficiency such that cleavage at -1 could not be detected and quantified.

The rate of cleavage (k_app_; Table 1) for *Pfu* CP RPR_wt_ (without protein) was ≈ 7-fold lower than for *Pfu* RPR_wt_ while determination of k_obs_ and K^sto^ revealed that both were affected ≈four- to five-fold, respectively, resulting in a 17-fold reduction in k_obs_/K^sto^ (Table 2). This is a significant lower reduction compared to the 500-fold drop in k_obs_/K^sto^ in response to deleting the S-domain in the *Eco* RPR system (Table 2; see also Refs [20, 23]).

We conclude that the S-domain is not essential for cleavage in the *Pfu* RPR alone reaction and as such supporting that the C-domain is responsible for catalysis also in the case of type A archaeal RNase P (see also Refs [11, 24, 32, 42]). However, the S-domain boosts the catalytic performance but to a lesser extent than for *Eco* RPR (see Discussion).

#### The absence of the S-domain or disruption of the P8/ P18-interaction affects the charge distribution at and in the vicinity of the cleavage site

A correct TSL/ TBS-interaction leads to efficient and correct cleavage [16, 19, 23]. Moreover, cleavage of pATSer derivatives in which the 2'OH at position -1 in the substrate had been replaced with 2'NH_2_ showed that the frequency of cleavage at -1 is reduced with increasing pH. Most likely this is because the 2'NH_2_ becomes protonated and positively charged with decreasing pH, thereby reducing cleavage at the canonical site +1 [38, 54]. The shift of the cleavage site is also dependent on the structural topography of the +1/+72 base pair in the substrate. We have argued that this is due to a change in the charge distribution at the cleavage site in the RPR-substrate complex ([37]; however, see Ref [55] for an alternative model). Hence, to test whether the absence of the S-domain and disruption of the P8/ P18-interaction influence the charge distribution/ protonation near the cleavage site in the RPR-substrate complex we studied the cleavage pattern of the pATSerUG variant pATSerU_am_G, in which the 2'OH was replaced with 2'NH_2_ at -1, at different pH (Fig 5; see Materials and methods).

**Fig 8:**
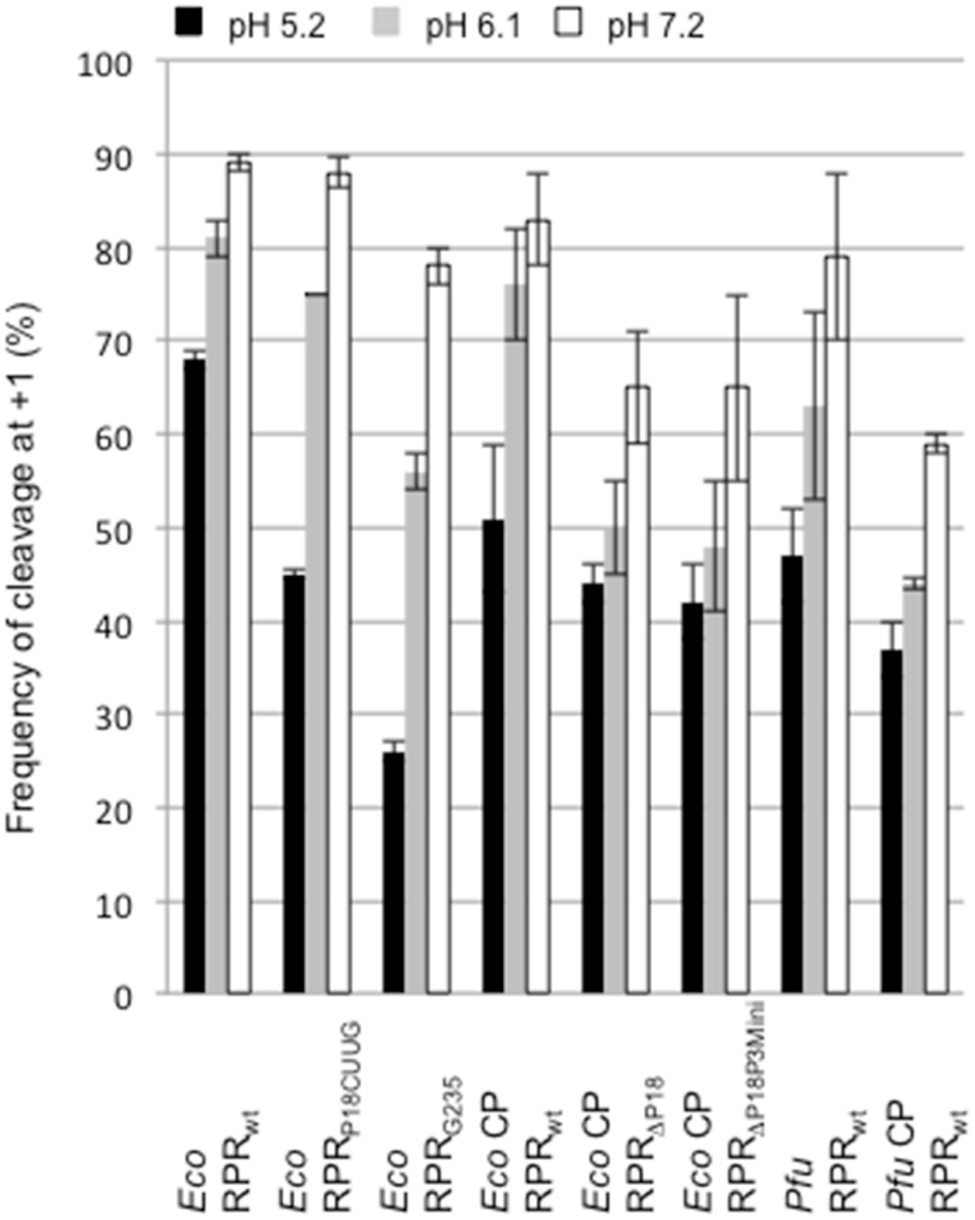
Frequency of cleavage of pATSerU_am_G at the +1 position with different RPRs at different pH as indicated. Reactions were done under single turnover (substrate concentration ≤0.02 μM) conditions at 37°C in buffer C at 800 mM Mg^2+^ as described elsewhere ([37, 38]; see also Materials and methods). The concentrations of RPRs were (reaction times in parenthesis): *Eco* RPR_wt_ 0.8 μM (10 min), *Eco* RPR_P18CUUG_ 0.8 μM (10 min), *Eco* CP RPR_wt_ 11 μM (270 min), *Eco* RPR_delP18_ 12 μM (270 min), *Eco* RPR_delP18P3Mini_ 14.5 μM (270 min), *Pfu* RPR_wt_ 4.5 μM (60 min) and *Pfu* CP RPR_wt_ 20 μM (270 min). The plots are based on four independent experiments and the bars indicate experimental errors.

The cleavage frequency of pATSerU_am_G at +1 increased with increasing pH for all the RPRs variants as expected from our previous data (Fig 8 and S3 Fig). However, compared to *Eco* RPR_wt_ and *Eco* CP RPR_wt_ the trend was that higher pH was required to reach 50% cleavage at +1 for the other RPR variants (including the *Pfu* RPR variants). For the all-ribo substrate pATSerUG the site of cleavage did not change with pH irrespective of RPR variant (S3 Fig).

We also inquired if a structural change in the TBS region (in the vicinity of where P18 contact P8; Fig 2B) in the S-domain affects cleavage of pATSerU_am_G as a function of pH differently compared to *Eco* RPR_wt_. Hence, we examined the cleavage pattern for *Eco* RPR_G235_ at different pH. This change in the RPR is known to influence cleavage site recognition (Fig 2B; [16, 19, 23]). Again a higher pH was needed to give 50% cleavage at the +1 position compared to *Eco* RPR_wt_ (Fig 8).

To conclude, we interpret these data to suggest that the S-domain, the P8/ P18-interaction and the structural topology of the TBS region influence the p*K*a of the 2'NH_2_ and/or the charge distribution at the cleavage site.

## Discussion

### Importance of the P6- and P8/ P18-interactions

The P6- and P8/ P18-interactions play important structural roles in folding the RPR where P6 is an intra C-domain interaction while P8/ P18 is involved in connecting the S- and C-domains (Fig 2B). However, information of their impact on the structure and function of RPRs lacking the S-domain, *e*.*g*. *Eco* CP RPR_wt_, is scarce. In this context, type T archaeal RPRs are equipped with a degenerated S-domain and secondary structure modeling suggests the presence of P6 but its contribution to catalysis has not been studied. As for *Pfu* RPR, P18 is also absent in type T RPRs [6, 12, 13]. Studying RNase H accessibility and cleavage of the model hairpin loop substrates pATSerUG we provide data suggesting that residues 5'G_276_C_277_C_278_C279 are likely engaged in pairing with residues 5'G_82_G_83_G_84_C_85_ in the absence of the S-domain (Fig 2A). We refer to this interaction as the "P6-mimic" and our data indicate that its presence contributes to the catalytic performance by *Eco* CP RPR. As such, our findings also provide support for the existence and functional importance of P6 in type T RPR. Moreover, deleting the S-domain in the type B *B*. *subtilis* RPR decreased the cleavage rate ≈25000-fold [21]. This is in contrast to the 120-fold reduction in the rate observed for the type A *Eco* RPR (Table 1; see also Refs [22, 23]). Type B lacks P6, however, recent data indicate that disruption of the intra-domain interaction between L5.1 and L15.1 in the C-domain affects both folding and the catalytic activity in a full-size RPR context [27]. Hence, it will be of interest to understand whether the L5.1/ L15.1 interaction (or a mimic) is present in the absence of the S-domain (see also Ref [21]). If this is the case we predict that it contributes to catalysis by the type B RPR lacking the S-domain.

In contrast to disruption of the "P6-mimic", removal of P18 did not result in any significant change in the catalytic performance for *Eco* CP RPR (Table 2). However, disruption of the P8/ P18-interaction, or deletion of P18, in full-size *Eco* RPR reduced cleavage of pATSerUG and affected the overall structure to a certain degree. While disrupting the P8/ P18 (P18 still present) influenced both the kinetic constants (k_obs_ and K^sto^; 24- and ≈10-fold change, respectively, compared to *Eco* RPR_wt_; Table 2) deleting P18 resulted in a dramatic decrease in k_obs_ (>3000-fold) in cleavage of the model substrate pATSerUG while no change in K^sto^ was detected. Given that K^sto^ ≈ K_d_ this might indicate that P18 interferes with binding of pATSerUG when its interaction with P8 is disrupted while this is not the case in its absence. Nevertheless, previous multiple turnover kinetic studies using pre-tRNA and pre-4.5S RNA reported that disruption of P8/ P18 or deletion of P18 affects binding affinity (K_m_) and k_cat_ in cleavage of pre-tRNAs with type A RPR with and without protein. The levels of change differ comparing our single turnover data and previously reported results ([28–30]; see below). This is likely due to different reaction conditions, choice of substrate and RPR (*Eco* RPR and *Thermus thermophilus*, *Tth*, RPR). In this context we also note that earlier data suggested that the impact of deleting P18 is suppressed by raising the ammonium concentration to 3 M [29]. We conclude that irrespective of substrate presence of P18 and the P8/ P18-interaction have an impact on the catalytic performance by bacterial type A RPR while in the absence of the S-domain (and the P8/ P18 interaction), as in *Eco* CP RPR, P18 has no significant impact on catalysis.

Calculating the ΔΔG using k_obs_/K^sto^ values [52] revealed that the contribution of the P8/ P18-interaction is between 3.1 and 5 kcal/mol for full-size *Eco* RPR while the contribution of the S-domain is approximately 3.8 kcal/mol (Table 2). Extracting and using the k_cat_/K_m_ (= k_obs_/K^sto^) values (for type A *Eco* and *Tth* RPRs; Table 2) from previous reports [28–30] to calculate the ΔΔG values suggest that the contribution of P18 and the P8/ P18-interaction varies between 1 and 1.8 kcal/mol. In the *Eco* RPR pre-tRNA-system without the C5 protein disruption of the P8/ P18-interaction resulted in a loss of 1.5 kcal/mol [30], which is two-fold lower than the value obtained using pATSerUG (Table 2). The reason for this discrepancy could be due to the difference in reaction conditions (*e*.*g*., here we used higher [Mg^2+^]) and/or the way the two substrates interact with the RPR where pre-tRNA has a structurally intact TSL-region. The importance of P18 in the full-size RPR context can be rationalized by its structural role in connecting the S- and C-domains and structurally orient these domains in a productive/ correct manner as discussed by Li et al. ([24]; Figs 2 and 3). This together with that a productive TSL/ TBS-interaction in the S-domain affects catalysis [16, 19, 23, 31, 34] opens for the possibility that the P8/ P18-interaction acts as a structural mediator in the "communication" between TSL/ TBS-interaction and the cleavage site leading to positioning of chemical groups and Mg^2+^ that result in correct and efficient cleavage. Consistent with this is that disruption of the P8/ P18-interaction, removal of the S-domain (and P18) as well as alteration in the vicinity of the structure were P18 connects (as in the *Eco* RPR_G235_ variant; Fig 2B; Ref [19]) seems to influence the charge distribution in the vicinity of the cleavage site (Fig 8). That the P8/ P18-interaction influences events at the cleavage site is also supported by data using a derivative of pATSerCG in which the loop had been replaced with a GAAA-tetra loop (pATSerCG_GAAA_; Fig 5). *Eco* RPR_wt_ cleaves this substrate preferentially at -1 (81±2%) and as we reported elsewhere this is most likely due to the absence of a productive/ correct TSL/ TBS-interaction [16, 19]. For *Eco* RPR_P18CUUG_ (disrupted P8/ P18-interaction) we observed a lower but reproducible cleavage (68±2%) at the alternative site -1 while absence of the S-domain (and the P8/ P18-interaction) results in cleavage preferentially at +1 [23]. Taken together, the type A *Eco* RPR S-domain and P8/ P18-interaction play important roles for the catalytic performance and site selection. Moreover, since removing P18 in *Eco* CP RPR did not affect cleavage (or the structure to any significant extent) it is likely that P18 itself does not influence catalysis but the P8/ P18-interaction does, consistent with that *Eco* RPR_delP18_ and *Eco* RPR_P18CUUG_ are poor catalysts in cleaving pATSerUG (Table 2). But noteworthy, the presence of P18 that cannot interact with P8 does affect pATSerUG binding and the reason to this is unclear (however see above). In this context, our unpublished structural probing data of full-size *Eco* RPR variants suggest that substitution of A248, which is positioned close to the cleavage site in the RNase P-tRNA structure [18], influence the structure of P18.

### Comparing the type A *Eco* and *Pfu* RPRs

Our data show that the *Pfu* RPR retained its catalytic activity upon removing the S-domain. Tsai *et al*. [6] provided data where they showed that a *Pfu* CP RPR construct is indeed catalytic however only in the presence of proteins. A rational for that they did not detect any cleavage activity without proteins might be differences in reaction conditions. We used a significantly higher Mg^2+^-concentration, which has been reported to increase low or unnoticed cleavage efficiency by *Eco* RPR variants [22, 34]. In addition, the choice of substrate differs in these two studies, pre-tRNA *vs*. pATSer, which might also be a factor. It should also be noted that our data are in accordance with that the activity of the archaeal type A *M*. *thermoautotrophicus* RPR substantially increases when its C-domain is linked to the *Eco* RPR S-domain [24].

Comparison of our current data, where we removed the S-domain of *Pfu* RPR_wt_, with our previous data [31] shows an effect on both the kinetic constant k_obs_ and K^sto^ in cleaving pATSerUG. This is similar to the situation for *Eco* RPR but the magnitude of change in k_obs_ for *Eco* RPR was higher (cf. 35-fold *vs*. 3.6-fold in the case of *Pfu* RPR; Table 2). Using the k_obs_/K^sto^ values for *Pfu* RPR_wt_ and *Pfu* CP RPR and calculating ΔΔG gives a loss of 1.7 kcal/mol as a result of removing the S-domain (Table 2). This should be compared to the 3.8 kcal/mol loss seen for *Eco* RPR_wt_ (see above). Full-size *Eco* RPR_wt_ can form a productive/ correct interaction with the substrate TSL-region while *Pfu* RPR_wt_ in the absence of proteins interacts differently with TSL [31]. Together this suggests that in the RPR alone reaction the *Pfu* S-domain plays a less important role in cleavage of the model hairpin loop substrate pATSerUG than in the *Eco* RPR case. Similar reduction in the rate (12-fold) was also observed upon removing the S-domain in a *cis* RPR-pre-tRNA construct based on the archaeal *M*. *jannaschii* type M system [42]. In this context, as reported previously the S-domain of type A archaeal RPR appears to hamper the activity in the RNA alone reaction [24, 32]. These authors also provided data where structural changes improved the activity of the type A archaeal *Methanothermobacter thermoautotrophicus* RPR and their findings are likely to be applicable to rationalize the difference in activity comparing *Eco* and *Pfu* RPRs.

Taken together, for *Pfu* RPR the influence of the S-domain is perhaps reflected by the fact that two of the five RNase P proteins, Rpp21 and Rpp29, bind to the S-domain and influence the interaction with the T-loop region of pATSer model substrates [31]. Moreover, considering the absence of P18 in archaeal type A RPRs it has been discussed that its role has been taken over by the Pop5 and Rpp30 proteins [24]. In this context we emphasize that removal of P18 in full-size *Eco* RPR reduced k_obs_ to a level ≈10-fold lower compared to k_obs_ as determined for *Pfu* RPR_wt_ and a loss of 5 kcal/mol relative to *Eco* RPR_wt_, one kcal/mol lower compared to *Pfu* RPR_wt_ Table 2; both *Eco* RPR_delP18_ and *Pfu* RPR_wt_ lack P18). To conclude, combined these data raise the question whether the evolution of a more complex RNase P in terms of the number of protein subunits is linked to a decrease in the contribution of the S-domain to catalysis see also Refs [24, 32].

## Supporting information

S1 Fig Cleavage of pATSerUG as a function of Mg^2+^ concentration. Cleavage by *Eco* CP RPR_delP18_ (A), *Eco* CP RPR_delP18P3Mini_ (B) and *Pfu* CP RPR_wt_ (C). Cleavage by *Eco* CP RPR_delP18_ (A), *Eco* CP RPR_delP18P3Mini_ (B) and *Pfu* CP RPR_wt_ (C). The experiment was performed in buffer C, 0.8 M NH_4_OAc (pH 6.1)at 37°C in the presence of indicated amount of Mg(OAc)_2_. The concentrations of RPRs ranged between 1 to 1.5 μg per μl and the substrate concentration was ≤0.02 μM. For the calculations, we used the 5' cleavage fragments and the data are the mean of three independent experiments. The bars indicate the experimental errors. For details see main text.
(TIFF)

S2 Fig Secondary structure model of the type A *Pfu* RPR. (TIF)

S3 Fig Cleavage of pATSerUG and pATSerU_am_G by *Eco* CP RPR_wt_, *Eco* CP RPR_delP18_, *Eco* CP RPR_delP18P3Mini_, *Pfu* CP RPR_wt_, *Eco* RPR_wt_ and *Eco* RPR_P18CUUG_. The experiment was performed at 37°C in buffer C, 0.8 M NH_4_OAc, 800mM Mg(OAc)_2_ at pH 5.2, 6.1 and 7.2 (the black triangles mark the increase in pH). The concentration of substrates were ≤0.02 μM while the concentration of RPRs were as indicated below: S = substrate, C1 and C2 = controls, no RPR added (1) and cleavage of pATSerUG with *Eco* RPR_wt_ 0.8 μM for 4 sec (2), 5'-L mark the cleavage 5' cleavage fragments due to cleavage at +1 and at -1. (A) and (B) [longer exposure of selected region shown in A] The concentrations of the RPRs and reaction times (in parenthesis) were: lane marked with C2 (4 sec), control (see above), *Eco* RPR_wt_ 0.8 μM (10 min), *Pfu* RPR_wt_ 4.5 μM (60 min), *Eco* CP RPR_wt_ 11 μM (270 min), *Eco* CP RPR_delP18_ 12 μM (270 min), *Eco* CP RPR_delP18P3Mini_ 14.5 μM (270 min) and *Pfu* CP RPR_wt_ 20 μM (270 min). (C) Cleavage of pATSerUG with RPRs as indicated (only the migration of 5' cleavage fragments are shown). Concentrations of the RPRs and reaction times (in parenthesis) were: lanes marked with C1 (60 min) and C2 (4 sec) controls (see above), *Eco* CP RPR_wt_ 13.7 μM (10 min), *Eco* CP RPR_delP18_ 12 μM (30 min), *Eco* CP RPR_delP18P3Mini_ 14.5 μM (30 min), *Pfu* CP RPR_wt_ 20.3 μM (60 min). (D) Cleavage of pATSerUG and pATSerU_am_G with RPRs as indicated (only the migration of 5' cleavage fragments are shown). Concentrations of the RPRs and reaction times (in parenthesis) were: control lanes marked with C1 (pATSerUG no RPR, 10 min), C2 (pATSerUG with 0.8 μM *Eco* RPR_wt_, 4 sec) and C3 (pATSerU_am_G no RPR, 10 min), *Eco* RPR_wt_ 0.8 μM (pATSerUG, 4 sec and pATSerU_am_G, 10 min), *Eco* RPR_P18CUUG_ 0.8 μM (pATSerUG, 1 min and pATSerU_am_G, 10 min) and *Pfu* RPR_wt_ 4.5 μM (10 min). For details see main text.
(TIFF)
