# Supplementary figures and images for: Critical domain interactions for type A RNase P RNA catalysis with and without the specificity domain
Source: PLoS One. 2018 Mar 6;13(3):e0192873. doi: 10.1371/journal.pone.0192873 (PMC5839562; doi:10.1371/journal.pone.0192873)

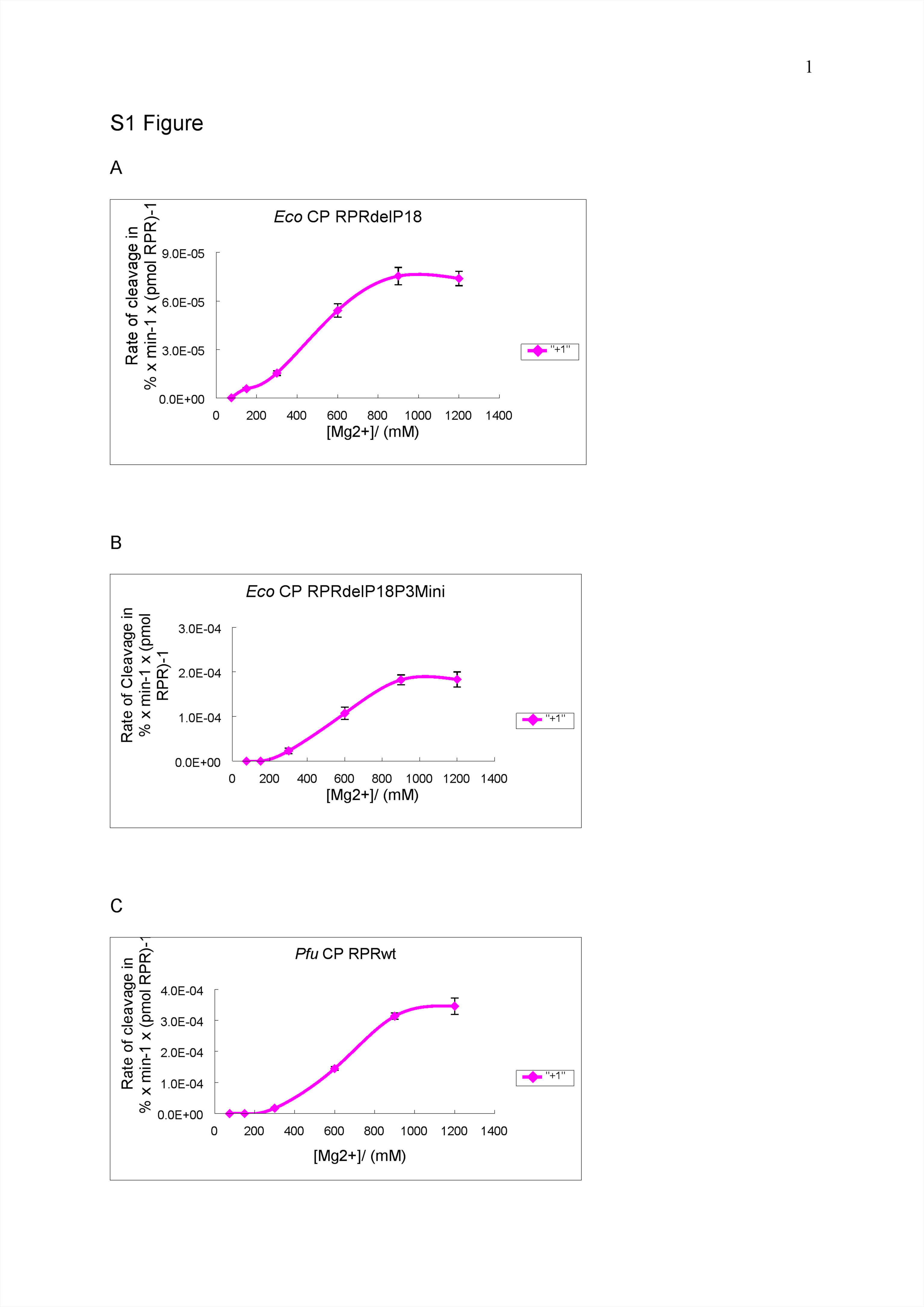

Supplement: S1 Fig — Cleavage by Eco CP RPRdelP18 (A), Eco CP RPRdelP18P3Mini (B) and Pfu CP RPRwt (C). Cleavage by Eco CP RPRdelP18 (A), Eco CP RPRdelP18P3Mini (B) and Pfu CP RPRwt (C). The experiment was performed in buffer C, 0.8 M NH4OAc (pH 6.1)at 37°C in the presence of indicated amount of Mg(OAc)2. The concentrations of RPRs ranged between 1 to 1.5 μg per μl and the substrate concentration was ≤0.02 μM. For the calculations, we used the 5' cleavage fragments and the data are the mean of three independent experiments. The bars indicate the experimental errors. For details see main text. (TIFF) [file pone.0192873.s001.tiff]

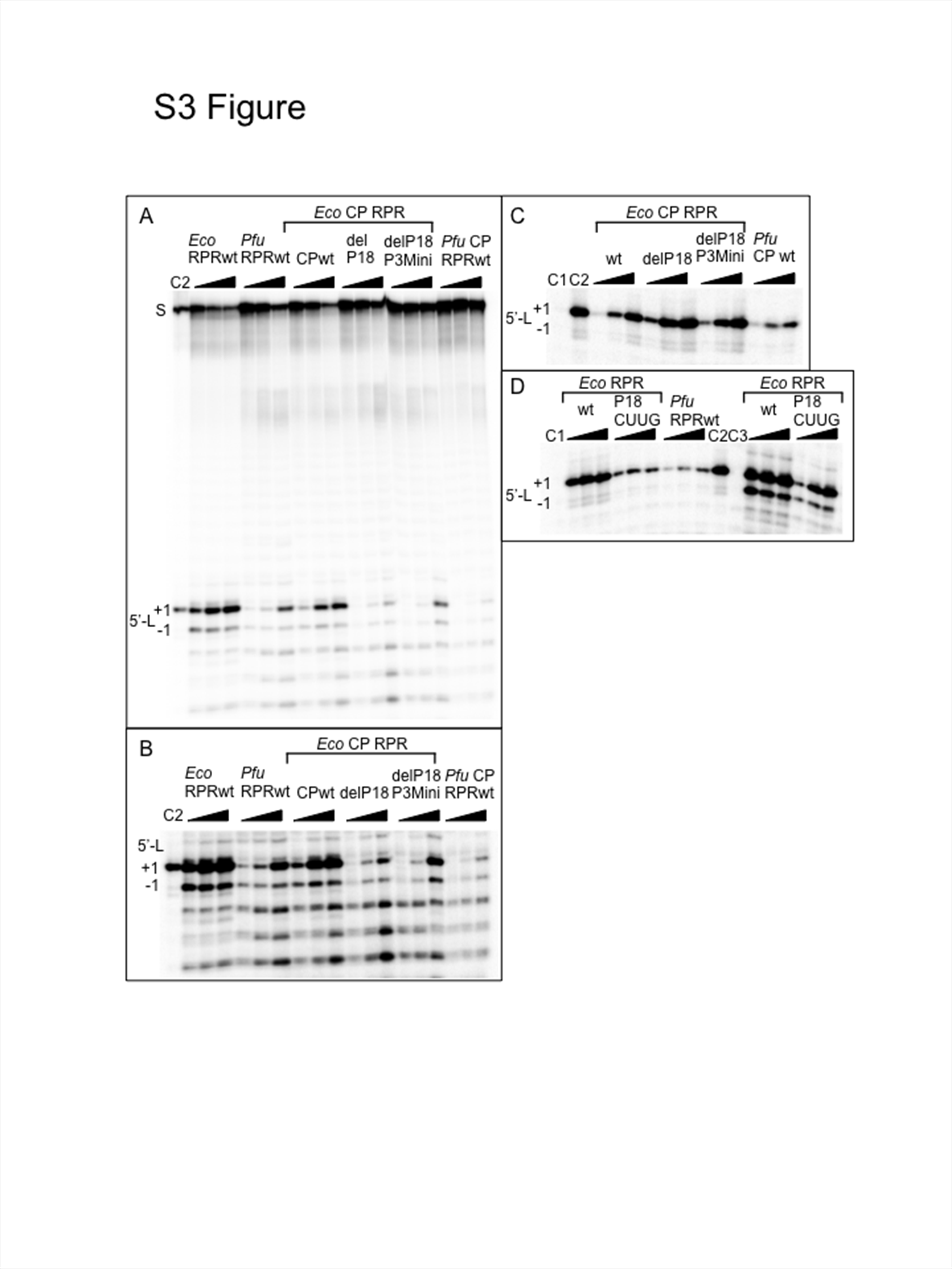

Supplement: S3 Fig — The experiment was performed at 37°C in buffer C, 0.8 M NH4OAc, 800mM Mg(OAc)2 at pH 5.2, 6.1 and 7.2 (the black triangles mark the increase in pH). The concentration of substrates were ≤0.02 μM while the concentration of RPRs were as indicated below: S = substrate, C1 and C2 = controls, no RPR added (1) and cleavage of pATSerUG with Eco RPRwt 0.8 μM for 4 sec (2), 5'-L mark the cleavage 5' cleavage fragments due to cleavage at +1 and at -1. (A) and (B) [longer exposure of selected region shown in A] The concentrations of the RPRs and reaction times (in parenthesis) were: lane marked with C2 (4 sec), control (see above), Eco RPRwt 0.8 μM (10 min), Pfu RPRwt 4.5 μM (60 min), Eco CP RPRwt 11 μM (270 min), Eco CP RPRdelP18 12 μM (270 min), Eco CP RPRdelP18P3Mini 14.5 μM (270 min) and Pfu CP RPRwt 20 μM (270 min). (C) Cleavage of pATSerUG with RPRs as indicated (only the migration of 5' cleavage fragments are shown). Concentrations of the RPRs and reaction times (in parenthesis) were: lanes marked with C1 (60 min) and C2 (4 sec) controls (see above), Eco CP RPRwt 13.7 μM (10 min), Eco CP RPRdelP18 12 μM (30 min), Eco CP RPRdelP18P3Mini 14.5 μM (30 min), Pfu CP RPRwt 20.3 μM (60 min). (D) Cleavage of pATSerUG and pATSerUamG with RPRs as indicated (only the migration of 5' cleavage fragments are shown). Concentrations of the RPRs and reaction times (in parenthesis) were: control lanes marked with C1 (pATSerUG no RPR, 10 min), C2 (pATSerUG with 0.8 μM Eco RPRwt, 4 sec) and C3 (pATSerUamG no RPR, 10 min), Eco RPRwt 0.8 μM (pATSerUG, 4 sec and pATSerUamG, 10 min), Eco RPRP18CUUG 0.8 μM (pATSerUG, 1 min and pATSerUamG, 10 min) and Pfu RPRwt 4.5 μM (10 min). For details see main text. (TIFF) [file pone.0192873.s003.tiff]
